# Regio- and Stereoselectivity of the Norrish–Yang Photocyclization of Dialkyl 1,2-Diketones: Solution versus Solid State Photochemistry of Two Polymorphs

**DOI:** 10.1021/acs.joc.2c01855

**Published:** 2022-10-25

**Authors:** Dimitri Alvarez-Dorta, Elisa I. León, Ángeles Martín, Alan R. Kennedy, Inés Pérez-Martín, Kenneth Shankland, Ernesto Suárez

**Affiliations:** † Síntesis de Productos Naturales, Instituto de Productos Naturales y Agrobiología del CSIC, Avda. Astrofísico Francisco Sánchez 3, 38206 La Laguna, Tenerife, Spain; ‡ WestCHEM Department of Pure and Applied Chemistry, University of Strathclyde, Glasgow G1 1XL, Scotland, United Kingdom; § School of Pharmacy, University of Reading, Reading RG6 6AD, United Kingdom

## Abstract

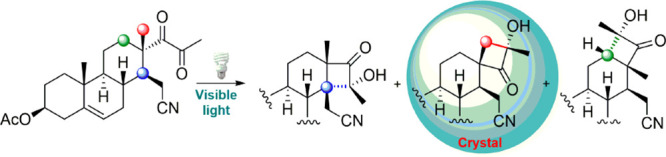

As shown by X-ray crystallography, crystals of 3β-acetoxy-16,17-seco-17,20-dioxopregn-5-ene-16-nitrile are dimorphic. The regioselectivity of the Norrish–Yang type II photocyclization under visible light of this steroidal 1,2-diketone, which bears primary, secondary, and tertiary nonequivalent abstractable γ-hydrogens, dramatically increases in the crystalline state of both polymorphs. X-ray crystallography and molecular mechanics calculations reveal crystal structure–solid state photochemistry relationships.

Norrish–Yang type II photocyclization is the almost exclusive photochemical reaction of 1,2-diketones (**I**, X = O) bearing abstractable γ-hydrogens after UV or visible light irradiation (Scheme 1).^1,2^ Thus, 1-hydroxy-2-cyclobutanones (**III**) are obtained from a highly regioselective 1,5-hydrogen atom transfer (1,5-HAT) reaction promoted by the excited carbonyl group and the subsequent Yang cyclization of the 1,4-biradical intermediate (**II**).^3^ These α-hydroxycyclobutanones readily undergo oxidative ring opening to form 4-oxo-acids (**IV**) in high yield.^4^ This tandem sequence (Norrish–Yang and oxidation) can be considered formally as a 1,3-stereocontrolled transference of an acyl group from the parent diketone^5^ and has been applied to the synthesis of a number of natural products with remarkable success.^6^

**Scheme 1:**
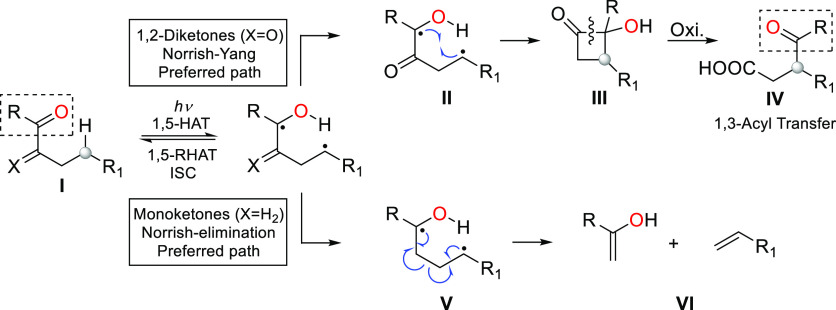
Norrish Type II Reactions of Mono- and 1,2-Diketones HAT: Hydrogen atom transfer; RHAT: Reversal hydrogen atom transfer; ISC: intersystem crossing.

The competitive Norrish II photoelimination reaction is a highly unusual process in 1,2-diketones, and only two examples had been described in the literature until our recently published work.^7^

Although the Norrish-elimination is the preferred reaction pathway in monoketones (**I**, X = H_2_) where the 1,4-diradical (**V**) undergoes further cleavage to produce alkenes and enols (**VI**), both Norrish type II processes, exclusively promoted by UV irradiation, have been intensively investigated on this functional group.^8^ In many cases the Norrish–Yang photoreaction of monoketones has been utilized in the key step of natural products synthesis.^2e,9^ Due to the reversibility of the initial transfer reaction, the final photoproducts do not always proceed from the most stereoelectronically favored abstraction.^10^

It is well-known that the photochemical behavior of organic compounds changes considerably on going from solution to solid state.^11^ Thus, enhanced regioselectivity has been observed in the Norrish–Yang photocyclization of crystalline monoketones, with a significant improvement in the formation of cyclobutanols when solid state photochemistry was employed instead of conventional solution photochemistry.^12^ Even a direct correlation between the crystal structure and their reactivity in solid state has been established.^10a,13^ The photochemistry of crystalline polymorphic monoketones has also been the subjects of several studies.^12e,14^ In contrast, there is a paucity of information on the Norrish–Yang photocyclization of crystalline 1,2-diketones, and there do not appear to be studies on the influence of the geometry on the regio- and stereoselectivity of this process.^15^ Neither have we found information related to the photochemistry of polymorphic 1,2-diketones. A detailed study on the stereo-, chemo-, and diastereoselectivity of a solid-state Norrish–Yang cyclization of a related ketoamide has recently been published.^15d^

Herein we report on the results obtained from a comparative study in solution and in solid state on the photocyclization under visible light irradiation of the alkyl 1,2-diketone **1** that we had previously obtained from a pregnane derivative by using a methodology developed in our laboratory (Scheme 2).^16^ This molecule, which retains the conformational restrictions inherent in a steroid, has three different types of potentially abstractable γ-hydrogen atoms: the primary hydrogens of the 18-Me group (pregnane numbering), the secondary hydrogens at C-12, and the tertiary hydrogen atom at C-14. All these structural features provide the most suitable environment to study the regio- and stereoselectivity of the Norrish–Yang photocyclization. In addition, the fact that it is a dimorphic crystalline product renders it an ideal model to determine structure–reactivity relationships.

**Scheme 2:**
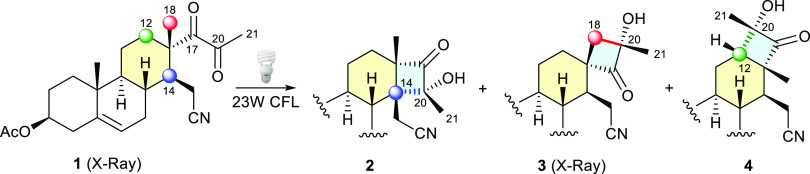
Photocyclization of Alkyl 1,2-Diketone **1** in Solution by Irradiation With a Philips master PL electronic daylight lamp 23 W/865.

The photochemistry of diketone **1** was first investigated in deoxygenated benzene by irradiation under direct sunlight at room temperature for 3 h (Table 1, entry 1). After purification by silica gel chromatography α*-*hydroxycyclobutanones **2** (66%, from H-14 abstraction), **3** (20%, from H-18 abstraction), and **4** (6%, from H-12 abstraction) were obtained in good overall yield (Scheme 2). Remarkably, only one diastereoisomer of each α-hydroxycyclobutanone was formed.

**Table 1:** Photocyclization of Alkyl 1,2-Diketone **1** in Solution and Crystalline State^a^

entry	medium	*T* (°C)	*t* (h)	**2**/**3**/**4**^b^	(%)^c^
1	C_6_H_6_^d^	r.t.	3	72/22/6	92
2	C_6_D_6_	25	3	74/19/7	88
3	CDCl_3_	25	2	78/20/2	81
4	CDCl_3_	–60	4	69/21/10	79
5	CH_3_CN	25	3.5	69/25/6	81
6	*t*BuOH	30	3.5	75/21/4	82
7	Et_2_O	25	2.5	77/17/4	83
8	Et_2_O	–40	4.5	80/15/5	81
9	Et_2_O	–90	4.5	83/13/4	84
10	[BMIm]OTf^e^	25	4	52/41/7	72
11	**1A** (crystals)	25	9	0/100/0	92
12	**1B** (crystals)	25	30	10/90/0	93

a Irradiation with a Philips master PL electronic daylight lamp 23 W/865.

b Ratio measured by ^1^H NMR spectroscopy.

c Chromatographic isolated yield.

d Irradiation with direct sunlight.

e [BMIm]OTf: 1-butyl-3-metylimidazolium trifluoromethanesulfonate.

The structure and stereochemistry of the isomeric photoproducts **2**–**4** were established on the basis of 1D and 2D NMR experiments [see Figures S1 and S2 in the Supporting Information (SI)]. As observed, the carbon substitution pattern of the three cyclobutanones is quite different [**2** (4 CH_3_, 7 CH_2_, 4 CH, 8 C); **3** (3 CH_3_, 8 CH_2_, 5 CH, 7 C); **4** (4 CH_3_, 6 CH_2_, 6 CH, 7 C)], and the ^13^C{^1^H} NMR spectra and their respective DEPT analyses provide sufficient information to easily distinguish them from one another.

The structure assigned to hydroxycyclobutanone **3** and the configuration of the new stereogenic center at C-20 were unambiguously confirmed by X-ray crystallographic analysis (see Figure S8 and Table S7 in the SI). Unfortunately, all attempts to obtain crystals suitable for X-ray analysis from the other α-hydroxycyclobutanones **2** and **4** failed completely. For both compounds NOE interactions between methyl hydrogens at C-21 and different hydrogens of the β-east side of the molecule are in concordance with the formation of hydroxycyclobutanones **2** and **4** as *cis-*fused rings, which allows us to assign tentatively the final configuration to the two new chiral centers (20*R*/14*R* and 20*S*/12*S*, respectively) generated in the cyclization (for more details, see Figure S1 in the SI).

Additionally we studied the photochemical behavior of diketone **1** in several common organic solvents by irradiation under visible light with a daylight lamp (Table 1, entries 2–10). Regardless of the reaction conditions, the same three hydroxycyclobutanones **2**–**4** were detected by ^1^H NMR, once again each as a single diastereomer.

The regioselectivity of the photocyclization reaction was not significantly affected by the polarity, protic or hydrogen bonding character of the solvents (entries 1–9).^3c,17^ Several experiments were even performed at lower temperatures, but no appreciable differences were detected (entries 3 and 4 or 7, 8, and 9). As it is known that the use of ionic liquids in radical reactions can affect the reactivity and dynamics of radicals,^18^ this photoreaction was carried out also in [BMIm]OTf.^19^ In this case, we observed a significant change in the photoproducts ratio although it is not synthetically useful (entry 10). The Norrish–Yang photocyclization reaction of 1,2-diketones, as far as we know, has not been previously described in ionic liquids.^20^

Then we moved on to study the photoreactivity of alkyl 1,2-diketone **1** in solid state. As determined by X-ray crystallography, this diketone can exist in two polymorphic forms: Polymorph **1A** prepared by slow evaporation of a solution of acetone–*n*-hexane at rt for 60 h and polymorph **1B** from a solution of EtOAc–*n*-hexane at 0 °C for 5 h (see Figures S6 and S7, and Table S6 in the SI).

Crystals of polymorph **1A**, crushed between two Pyrex slides, were irradiated under visible light to provide exclusively **3**. Under these conditions, none of the other five theoretical possible isomers were detected by ^1^H NMR analysis of the crude reaction residue (entry 11). Although a higher regioselectivity has generally been found on the Yang photocyclization of monoketones in crystalline state than in solution,^12^ such a dramatic increase as this had not previously been observed either in monoketones or 1,2-diketones. In the photocyclization of polymorph **1B**, the formation of **3** predominates (90%), but a small amount of cyclobutanone **2** (10%) is also present in the reaction mixture (entry 12). The regio- and stereoselectivity observed in the high yielding photocyclization of polymorph **1A** in the solid state encouraged us to investigate whether we are dealing with a crystal-to-crystal transformation. Initially we investigated whether a single-crystal-to-single-crystal (SCSC) process was possible, as has occasionally been observed in the Norrish–Yang photocyclization of monoketones.^21^ However, irradiation of large crystals of polymorph **1A** caused cracking and breaking of the initial crystalline form into small fragments. Powder X-ray diffraction (PXRD) data confirm a microcrystalline structure for these fragments in excellent agreement with the single X-ray diffraction (SXRD) data of compound **3**, indicative of the expected transformation (see Figures S9, S10, S13, and S14 in the SI). A similar result has been observed during the UV photocyclization of a monoketone derivative (2,4,6-triisopropylbenzophenone),^21c^ and a possible explanation has been proposed. Since the photoreaction is initiated at the surface of the crystal, the strain generated inside causes the crack and break of the crystal lattice and the formation of microcrystals. In this sense, the reaction of polymorph **1A** can be considered as a crystal-to-crystal transformation (see Figures S9 and S10 in the SI). The analogous reaction of polymorph **1B** proceeded with crystal discoloration but without apparent change in their morphology to give an amorphous solid whose PXRD data show no evidence of a diffraction pattern (see Figures S11 and S12 in the SI).

To explain these photochemical results we must keep in mind that the viability of the initial 1,5-HAT reaction in Norrish type II processes is strongly dependent on the conformation of the TS (see Figure S3 in the SI). For this reason, in the restricted conformational mobility of the crystalline state the distance (*d*) between the excited carbonyl oxygen atom and the γ-hydrogen to be abstracted is of special importance and must be close to 2.72 Å (sum of the van der Waals radii).^12e,13^ In the three cases of Norrish Type II abstraction promoted by crystalline 1,2-diketones where *d* values have been calculated, these are in the range 2.4–2.69 Å.^7a,15^

A conformational analysis based in molecular mechanics calculations revealed that 1,2-diketone **1** adopts in solution two minimum energy staggered conformations by rotation around the C13–C17 single bond (Figure 1, see Tables S1, S2, and S3 in the SI for details).^22^ The two conformers are almost isoenergetic, where the global minimum *syn*-conformer **A** (Figure 1a, dihedral C18–C13–C17–O = 20°) is only about 0.5 kcal mol^–1^ more stable than the *anti*-conformer **B** (Figure 1b, dihedral C18–C13–C17–O = 141°) (see Figure S5 in the SI). Furthermore, the X-ray diffraction study established that both polymorphs **1A**,**B** adopt in the crystalline state an *anti*-conformation (dihedral C18–C13–C17–O = 148.17(13)°, and 142.3(2)° respectively) very similar to that present in conformer **B** (see Figures S6 and S7, and Tables S4 and S5 in the SI).

**Figure 1:**
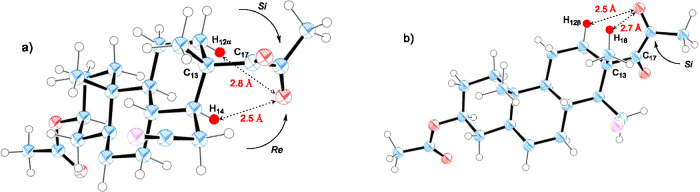
Solution conformational isomers of 1,2-diketone **1**. (a) Conformer **A** (Dihedral C18–C13–C17–O = 20°). (b) Conformer **B** (Dihedral C18–C13–C17–O = 141°). X-ray structures are very similar to conformer **B** (Dihedral C18–C13–C17–O = 148.17(13)° for polymorph **1A** and 142.3(2)° for polymorph **1B**).

With regard to the photoreaction in solution, it appears clear that diketone **1** reacts through both conformations in equilibrium, which leaves all potentially abstractable γ-hydrogens involved (tertiary axial H14 and secondary axial H12α from conformer **A** and primary H18 and secondary equatorial H12β from conformer **B**) at a convenient distance (*d* = 2.4–2.7 Å) for the 1,5-HAT reaction to take place (see Tables S1 and S2 in the SI). The fact that **2** is the main photoproduct obtained in all cases suggests that conformer **A** is the preferred, as expected. The use of an ionic liquid as solvent seems to affect this conformational equilibrium, favoring a greater participation of conformer **B**. In any case, the final photocyclobutanones **2**, **3**, and **4** ratio does not follow a reactivity pattern determined by the expected stability of the radical intermediate (tertiary ≫ secondary > primary) and seems rather the result of a complex distribution of the three 1,4-biradicals between cyclization and the reversal hydrogen atom transfer reaction (RHAT).

On the basis of the crystalline geometrical parameters of polymorph **1A** (see Table S4 in the SI), we could expect in solid state the initial abstraction of γ-hydrogens H18 (*d* = 2.5 Å) and H12β (*d* = 2.6 Å) (see Figure S6 in the SI). In the conformationally restricted crystal environment, the direct cyclization of the biradical generated from the H12β abstraction would lead to a highly energetic *trans*-fused cyclobutanone. In consequence the starting 1,2-diketone is regenerated from the RHAT reaction and the photochemical cyclization occurs exclusively from the H18 abstraction and the final formation of **3** as the sole product of the reaction. The loss of regioselectivity observed in the case of polymorph **1B** may be rationalized in terms of fewer restrictions in the environment of the methyl 1,2-diketone group during the photochemical reaction allowing the rotation about C13–C17 single bond since, as previously commented, is a relatively low-energy process.

Finally, the diastereoselectivity of the reaction with the exclusive formation of a single isomer of the hydroxylmethyl grouping at C-20 can also be explained. In the crystalline conformation, the 1,4-biradical generated from the diastereotopic *Si*-face of the excited carbonyl rapidly collapses to give the 20*S* isomer of **3** exclusively. This feature that has been called *retention of configuration at the carbonyl carbon* is generally observed in the solid state photocyclization of monoketones.^12d^ The formation of the 20*R* isomer would imply an attack by the *Re*-face of the carbonyl, which requires topochemically prohibited bond rotations in the crystal lattice.

The rapid collapsing of the 1,4-biradical can also be postulated as an explanation for the diastereoselectivity observed in solution during the formation of compounds **2** and **4**. From the conformer **A**, the cyclization of the 14,20-biradical by the *Re*-face leads exclusively to **2** with a 20*R* stereochemistry while the *Si*-facial adduct **4** with a 20*S* configuration is obtained from the cyclization of the 12,20-biradical also with absolute diastereoselectivity.

In summary this work shows that Norrish–Yang photocyclization of alkyl 1,2-diketones with several nonequivalent abstractable γ-hydrogens can be rendered completely regioselective passing from the solution to crystalline state. The regio- and stereoselectivity of the photoreaction in the solid state seems to be governed by geometrical parameters in a manner similar to the photocyclization of monoketones.

## Experimental Section

### General Information

Melting points were determined with a hot-stage apparatus. Optical rotations were measured at the sodium line at ambient temperature in CHCl_3_ solutions. IR spectra were measured in CHCl_3_ solutions. ^1^H NMR spectra were determined at 400 or 500 MHz in CDCl_3_ or C_6_D_6_. Chemical shifts are reported in parts per million (ppm) and are calibrated to residual solvent peaks (CHCl_3_ 7.26 ppm and C_6_H_6_ 7.15 ppm). ^13^C{^1^H} NMR spectra were determined at 100.4 or 125.7 MHz in CDCl_3_. Chemical shifts are reported in parts per million (ppm) and are calibrated to residual solvent peaks (CHCl_3_ 77.0 ppm). NMR peaks assignments and stereochemistries have been established using COSY, DEPT, HMBC, HSQC, and NOESY experiments. Low and high resolution mass spectra were recorded by using EI (70 eV). All single crystal diffraction measurements were performed at 123(2) K with a Oxford Diffraction Gemini instrument utilizing Cu radiation. Selected crystallographic parameters are given in the SI. PXRD was carried out using a Bruker D8 Advance diffractometer equipped with a LynxEye detector and monochromatic Cu Kα1 (λ = 1.54056 Å) radiation, operating in transmission capillary mode. Powdered samples were filled in 0.5 mm borosilicate glass capillaries, and data were initially collected over an angular range of 4 to 70° 2θ with a step size of 0.017°. Merck silica gel 60 PF (0.063–0.2 mm) was used for column chromatography. Circular layers of 1 mm of Merck silica gel 60 PF_254_ was used on a Chromatotron for centrifugally assisted chromatography. Elemental analyses were performed by the Microanalytical Service of the Institute. Commercially available reagents and solvents were analytical grade or were purified by standard procedures prior to use. The chloroform used in the photoreaction was purified by passing through a column of K_2_CO_3_ in the dark before use, but the utilization of strictly deoxygenated solvents does not seem to be necessary. The spray reagents for TLC analysis were conducted with 0.5% vanillin in H_2_SO_4_–EtOH (4:1) and further heating until development of color. The light source was a daylight Philips lamp (master PL electronic, 23 W/865) (for a description of the photoreaction equipment, see Figure S4 in the SI).

### 3β-Acetoxy-16,17-seco-17,20-dioxopregn-5-ene-16-nitrile (**1**)

Prepared as previously described by alkoxyl radical β-fragmentation of 3β-acetoxy-16β-azido-17α-hydroxy-preg-5-en-20-one.^16^ The product was isolated by silica gel column chromatography (benzene–EtOAc, 93:7) as bright yellow crystals (51%). [α]_D_ −58.7 (*c* = 1.46, CHCl_3_). ^1^H NMR (400 MHz, CDCl_3_) δ_H_ 5.37 (m, 1H), 4.59 (m, 1H), 2.34 (s, 3H), 2.02 (s, 3H), 1.33 (s, 3H), 1.03 ppm (s, 3H). ^13^C{^1^H} NMR (100.4 MHz, CDCl_3_) δ_C_ 206.2 (C), 200.6 (C), 170.4 (C), 139.3 (C), 121.0 (CH), 118.7 (C), 73.4 (CH), 50.1 (C), 48.4 (CH), 41.9 (CH), 37.7 (CH_2_), 36.7 (C), 36.6 (CH_2_), 35.5 (CH_2_), 31.8 (CH), 31.3 (CH_2_), 27.5 (CH_2_), 26.8 (CH_3_), 21.3 (CH_3_), 19.2 (CH_2_), 19.1 (CH_3_), 18.0 (CH_2_), 14.3 ppm (CH_3_). IR (CHCl_3_) ν = 2248, 1722 cm^–1^. MS (EI, 70 eV) *m*/*z* (%) = 342 (1) [M – COCH_3_]^+^, 325 (11), 254 (100), 213 (10). HRMS (EI) *m*/*z* [M–COCH_3_]^+^ calcd for C_21_H_28_NO_3_ 342.2069, found 342.2071. Anal. calcd for C_23_H_31_NO_4_: C, 71.66; H, 8.11; N, 3.63. Found: C, 71.84; H, 8.10; N, 3.63.

### Polymorph **1A**

Bright yellow crystals, mp 121–121.5 °C (slow evaporation of a solution of acetone–*n*-hexane at rt for 60 h); Crystal data and structure refinement: C_23_H_31_NO_4_, M_τ_ = 385.49, monoclinic, space group *P*2_1_, *a* = 9.5769(1) Å, *b* = 9.8814(1) Å, *c* = 10.6257(1) Å, β = 93.098(1)°, *V* = 1004.074(17) Å^3^, *Z* = 2, ρ_calcd_ = 1.275 Mg/m^3^, μ(Cu_Kα_) = 1.54180 Å, *F*(000) = 416, *T* = 123 (2) K, yellow crystal, 0.34 × 0.28 × 0.10 mm^3^, collected reflections 13014. The structure was solved by direct method, all hydrogen atoms were refined anisotropically using full-matrix least-squared based *F*^2^ to give R_1_ = 0.0316, *w*R_2_ = 0.0850 for 3655 independently observed reflections (|*F*_o_| > 2σ(|*F*_o_|)) and 257 parameters.

A small amount of polymorph **1A** was ground to a fine powder and loaded into a 0.5 mm capillary, and powder X-ray diffraction (PXRD) data were collected. The data were in excellent agreement with the single crystal X-ray diffraction (SXRD) structure.

### Polymorph **1B**

Bright yellow crystals, mp 116.3–116.8 °C (slow evaporation of a solution of EtOAc–*n*-hexane at 0 °C for 5 h); Crystal data and structure refinement: C_23_H_31_NO_4_, M_τ_ = 385.49, monoclinic, space group *P*2_1_, *a* = 7.6474(3) Å, *b* = 15.4448(5) Å, *c* = 9.2459(3) Å, β = 111.210(4)°, *V* = 1018.08(7) Å^3^, *Z* = 2, ρ_calcd_ = 1.257 Mg/m^3^, μ(Cu_Kα_) = 1.54180 Å, *F*(000) = 416, *T* = 123 (2) K, yellow crystal, 0.30 × 0.25 × 0.05 mm^3^, collected reflections 4100. The structure was solved by direct method, all hydrogen atoms were refined anisotropically using full-matrix least-squared based *F*^2^ to give R_1_ = 0.0382, *w*R_2_ = 0.1028 for 3103 independently observed reflections (|*F*_o_| > 2σ(|*F*_o_|)) and 257 parameters.

A small amount of polymorph **1B** was ground to a fine powder and loaded into a 0.5 mm capillary, and PXRD data were collected. The data were in excellent agreement with the SXRD structure.

### Photocyclization of 3β-Acetoxy-16,17-seco-17,20-dioxopregn-5-ene-16-nitrile (**1**)

**Method A** (Solution, irradiation in C_6_H_6_): A deoxygenated solution of diketone **1** (50 mg, 0.13 mmol) in dry C_6_H_6_ (10 mL), placed in a Schlenk tube, was irradiated with sunlight at room temperature. The photoreaction was periodically monitored by TLC analysis showing complete disappearance of starting diketone in 3 h. The reaction mixture was concentrated under reduced pressure and the crude residue purified by silica gel Chromatotron chromatography (hexanes–EtOAc, 85:15) to give **2** (33 mg, 0.09 mmol, 66%), **3** (9.8 mg, 0.03 mmol, 20%), and **4** (3 mg, 0.008 mmol, 6%).

**Method B** (Solution, general procedure for irradiation in common organic solvents): A deoxygenated solution of diketone **1** (5 mg, 0.013 mmol) in the corresponding dry solvent (0.6 mL), placed in a resonance tube, was irradiated with a daylight-lamp (Philips master PL electronic, 23 W/865). Progress of the reaction was monitored until completion by TLC. The solvent, temperature, time of irradiation, and hydroxycyclobutanones ratio and yields as established from ^1^H NMR spectroscopy and chromatography are specified in Table 1.

**Method C** (Solution, irradiation in ionic liquid): The IL [BMIm][OTf] (1-butyl-3-metylimidazolium trifluoromethanesulfonate)^18^ (70 mg) was taken in a vial and a solution of diketone **1** (5 mg, 0.013 mmol) in dry CH_2_Cl_2_ was added. After evaporation of CH_2_Cl_2_ under reduced pressure the resulting oil was extended as a film over the vial wall and irradiated under nitrogen with a daylight-lamp (Philips master PL electronic, 23 W/865) at 25 °C. When the yellow color of the reaction mixture completely faded (3 h), it was extracted twice with Et_2_O and the IL residues were removed with ultrasound in a water bath. After evaporation of the solvent, the crude was analyzed by ^1^H NMR and purified by chromatography (hexanes–EtOAc, 80:20) to give (3.6 mg, 0.0094 mmol, **2**/**3**/**4**, 52:41:7, 72%).

**Method D** (Solid-state, irradiation of single crystals): Crystals of polymorph **1A** (12.8 mg, 0.0333 mmol) were placed in a resonance tube and irradiated (Philips master PL electronic, 23 W/865) at 25 °C for 9 h. The reaction proceeded with cracking and breaking of the initial single crystalline form to give pure **3** as a polycrystalline solid (11.8 mg, 0.0306 mmol, 92%), which was analyzed by ^1^H NMR and purified by chromatography (hexanes–EtOAc, 80:20).

Analogously, for crystals of polymorph **1B** (11.5 mg, 0.0299 mmol) under irradiation (Philips master PL electronic, 23 W/865) at 25 °C for 30 h, the reaction proceeded with crystal discoloration but without an apparent change in their morphology to give an amorphous solid as a mixture of **2** and **3** (10.7 mg, 0.0278 mmol, 10:90, 93%), which was analyzed by ^1^H NMR and purified by chromatography (hexanes–EtOAc, 80:20).

**Method E** (Solid-state, irradiation of crushing crystals): The procedure consisted of crushing crystals of polymorph **1A** (2 mg, 0.0052 mmol) between two Pyrex microscope slides, taping the plates together, sealing the resulting “sandwich” under nitrogen in a polyethylene bag, and irradiating the ensemble on both sides with a daylight-lamp (Philips master PL electronic, 23 W/865) at 25 °C for 3 h, until the yellow color faded. ^1^H NMR spectroscopy of the reaction crude showed complete conversion of diketone **1A** exclusively into the hydroxycyclobutanone **3** (1.9 mg, 0.0049 mmol, 94%). This proceeded analogously for polymorph **1B** (2.5 mg, 0.0065 mmol), which after irradiation (Philips master PL electronic, 23 W/865) at 25 °C for 10 h gave a mixture of hydroxycyclobutanones **2** and **3**, which was purified by chromatography (hexanes–EtOAc, 80.20) to give (2.3 mg, 0.006 mmol, 10:90, 92%).

Compound **2**: Crystalline solid, mp 176–178 °C (slow evaporation of a solution of *n*-hexane–EtOAc at 0 °C). [α]_D_ +83 (*c* = 0.21, CHCl_3_). ^1^H NMR (500 MHz, CDCl_3_) δ_H_ 5.43 (m, 1H), 4.62 (m, 1H), 2.75 (d, *J* = 16.8 Hz, 1H), 2.31 (d, *J* = 16.8 Hz, 1H), 2.03 (s, 3H), 1.28 (s, 3H), 1.26 (s, 3H), 1.03 ppm (s, 3H). ^1^H NMR (500 MHz, C_6_D_6_) δ_H_ 5.28 (m, 1H), 4.73 (m, 1H), 2.45 (ddd, *J* = 13.1, 5.1, 2.5 Hz, 1H), 1.91 (d, *J* = 16.8 Hz, 1H), 1.74 (s, 3H), 1.13 (s, 3H), 0.84 (s, 3H), 0.76 ppm (s, 3H). ^13^C{^1^H} NMR (125.7 MHz, CDCl_3_) δ_C_ 214.3 (C), 170.8 (C), 139.5 (C), 121.8 (CH), 118.0 (C), 91.7 (C), 73.8 (CH), 62.7 (C), 45.5 (C), 44.9 (CH), 37.83 (C), 37.78 (CH_2_), 36.5 (CH_2_), 34.6 (CH), 32.0 (CH_2_), 27.5 (CH_2_), 27.4 (CH_2_), 23.3 (CH_2_), 21.4 (CH_3_), 20.1 (CH_2_), 19.9 (CH_3_), 18.1 (CH_3_), 16.9 ppm (CH_3_). IR (CHCl_3_) ν = 3581, 2248, 1775, 1725 cm^–1^. MS (EI; 70 eV) *m*/*z* (%) = 385 (<1) [M]^+^, 345 (3). HRMS (EI) *m*/*z* [M]^+^ calcd for C_23_H_31_NO_4_ 385.2253, found 385.2228. Anal. calcd for C_23_H_31_NO_4_: C, 71.66; H, 8.11; N, 3.63. Found: C, 71.63; H, 8.13; N, 3.67.

Compound **3**: Crystalline solid, mp 185–188 °C (slow evaporation of a solution of *n*-hexane–EtOAc at 0 °C). [α]_D_ −65 (*c* = 0.77, CHCl_3_). ^1^H NMR (500 MHz, CDCl_3_) δ_H_ 5.38 (m, 1H), 4.60 (m, 1H), 2.72 (dd, *J* = 13.2, 1.6 Hz, 1H), 2.58 (dd, *J* = 18.0, 6.1 Hz, 1H), 2.18 (dd, *J* = 18.0, 2.7 Hz, 1H), 2.10 (d, *J* = 13.3 Hz, 1H), 2.03 (s, 3H), 1.45 (s, 3H), 1.01 ppm (s, 3H). ^13^C{^1^H} NMR (100.4 MHz, CDCl_3_) δ_C_ 216.5 (C), 170.5 (C), 139.6 (C), 120.8 (CH), 118.1 (C), 85.8 (C), 73.5 (CH), 65.9 (C), 48.5 (CH), 40.7 (CH), 37.7 (CH_2_), 36.8 (CH_2_), 36.7 (C), 36.6 (CH_2_), 34.9 (CH_2_), 32.2 (CH), 30.7 (CH_2_), 27.5 (CH_2_), 21.4 (CH_3_), 21.0 (CH_3_), 20.3 (CH_2_), 19.1 (CH_3_), 17.8 ppm (CH_2_). IR (CHCl_3_) ν = 3585, 2256, 1774, 1728 cm^–1^. MS (EI; 70 eV) *m*/*z* (%) = 385 (2) [M]^+^, 325 (26). HRMS (EI, 70 eV) *m*/*z* [M]^+^ calcd for C_23_H_31_NO_4_ 385.2253, found 385.2278. Anal. calcd for C_23_H_31_NO_4_: C, 71.66; H, 8.11; N, 3.63. Found: C, 71.87; H, 8.14; N, 3.61. Crystal data and structure refinement for compound **3**: C_23_H_31_NO_4_, M_τ_ = 385.49, orthorhombic, space group *P*2_1_2_1_2_1_, *a* = 7.7874(13), *b* = 9.0718(19), *c* = 28.882(6) Å, β = 90°, *V* = 2040.4(7) Å^3^, *Z* = 4, ρ_calcd_ = 1.255 Mg/m^3^, μ(Cu_Kα_) = 1.54180 Å, *F*(000) = 832, *T* = 123 (2) K, colorless crystal, 0.28 × 0.20 × 0.05 mm^3^, collected reflections 10623. The structure was solved by direct method, all hydrogen atoms were refined anisotropically using full-matrix least-squared based *F*^2^ to give R_1_ = 0.0492, *w*R_2_ = 0.0856 for 3627 independently observed reflections (|*F*_o_| > 2σ(|*F*_o_|)) and 260 parameters.

PXRD data from the exposed (6500 K light for in excess of 30 h) powder sample of polymorph **1A** showed excellent agreement with the SXRD structure of compound **3**, indicative of the expected transformation. A single crystal of polymorph **1A** transforms, upon the light irradiation (6500 K light for in excess of 30 h), into a microcrystalline powder with disintegration of the initial single crystalline form.

PXRD data from the exposed (6500 K light for in excess of 30 h) powder sample of polymorph **1B** showed no crystalline diffraction, indicative of a loss of crystallinity. A single crystal of polymorph **1B** exposed (6500 K light for in excess of 30 h) was remounted and scanned on the diffractometer but yielded no diffraction spots, despite looking essentially identical (in morphology) to the crystal before exposure.

Compound **4**: Amorphous white solid, [α]_D_ −34 (*c* = 0.2, CHCl_3_). ^1^H NMR (500 MHz, CDCl_3_) δ_H_ 5.37 (m, 1H), 4.59 (m, 1H), 3.04 (dd, *J* = 7.4, 1.6 Hz, 1H), 2.57 (dd, *J* = 17.6, 6.1 Hz, 1H), 2.51 (dd, *J* = 17.7, 3.1 Hz, 1H), 2.03 (s, 3H), 1.48 (s, 3H), 1.43 (s, 3H), 1.0 ppm (s, 3H). ^1^H NMR (500 MHz, C_6_D_6_) δ_H_ 5.09 (ddd, *J* = 4.9, 2.1, 2.1 Hz, 1H), 4.74 (dddd, *J* = 11.4, 11.4, 4.7, 4.7 Hz, 1H), 2.46 (ddd, *J* = 13.2, 5.0, 2.4 Hz, 1H), 2.00 (dd, *J* = 17.4, 3.4 Hz, 1H), 1.95 (dd, *J* = 17.6, 6.3 Hz, 1H), 1.75 (s, 3H), 0.98 (s, 3H), 0.91 (s, 3H), 0.66 ppm (s, 3H). ^13^C{^1^H} NMR (125.7 MHz, CDCl_3_) δ_C_ 209.9 (C), 170.5 (C), 139.1 (C), 121.0 (CH), 119.7 (C), 89.6 (C), 73.3 (CH), 57.5 (CH), 44.6 (CH), 41.0 (CH), 38.7 (C), 37.7 (CH_2_), 36.6 (C), 36.4 (CH_2_), 30.8 (CH_2_), 29.6 (CH), 27.5 (CH_2_), 21.4 (CH_3_), 20.3 (CH_3_), 19.2 (CH_3_), 18.3 (CH_2_), 17.5 (CH_2_), 15.6 ppm (CH_3_). IR (CHCl_3_) ν = 3586, 2246, 1775, 1727 cm^–1^. MS (EI; 70 eV) *m*/*z* (%) = 385 (<1) [M]^+^, 325 (22). HRMS (EI; 70 eV) *m*/*z* [M]^+^ calcd for C_23_H_31_NO_4_ 385.2253, found 385.2226. Anal. calcd for C_23_H_31_NO_4_: C, 71.66; H, 8.11; N, 3.63. Found: C, 71.85; H, 8.22; N, 3.61.

## Supporting Information Available

Most significant NOESY and HMBC correlations for compounds **2**–**4**; geometrical parameters of conformers and polymorphs of compound **1**; crystal data and structure refinement for compounds **1A**, **1B**, and **3**; powder X-ray diffraction patterns before and after irradiation of polymorph **1A** and **1B**; copies of the ^1^H and ^13^C{^1^H} NMR spectra of all new compounds (PDF)
