# Supplementary material for: Regio- and Stereoselectivity of the Norrish–Yang Photocyclization of Dialkyl 1,2-Diketones: Solution versus Solid State Photochemistry of Two Polymorphs
Source: J Org Chem. 2022 Oct 25;87(21):14940–7. doi: 10.1021/acs.joc.2c01855 (PMC9639068; doi:10.1021/acs.joc.2c01855)

Supporting Information

Regio- and Stereoselectivity of the Norrish-Yang photocyclization of dialkyl 1,2-diketones. Solution versus solid state photochemistry of two polymorphs

Dimitri Alvarez-Dorta,[a] Elisa I. León,[a]* Ángeles Martín,[a] Alan R. Kennedy,[b] Inés Pérez-Martín,[a] Kenneth Shankland,[c] and Ernesto Suárez[a]*

[a] Síntesis de Productos Naturales, Instituto de Productos Naturales y Agrobiología del CSIC, Avda. Astrofísico Francisco Sánchez 3, 38206, La Laguna, Tenerife, Spain

[b] WestCHEM Department of Pure and Applied Chemistry, University of Strathclyde , Glasgow G1 1XL, Scotland, United Kingdom

[c] School of Pharmacy, University of Reading, Reading, RG6 6AD, United Kingdom

Table of Contents

1. Figure S1. Most Significant NOESY Correlations for Compounds **2–4** S3
2. Figure S2. Most Significant HMBC Correlations for Compounds **2–4** S3
3. Figure S3. Definition of Geometrical Parameters *D*, *d*, ω, Δ, and θ S3
4. Figure S4. Photoreactor description S3
5. Table S1. Geometrical Parameters of 1,2-Diketone **1**, Conformer **A** S4
6. Table S2. Geometrical Parameters of 1,2-Diketone **1**, Conformer **B** S4
7. Table S3. Coordinated Scan Calculation of C18–C13–C17–O Dihedral in 1,2-Diketone **1** .. S5–S6
8. Figure S5. Coordinated Scan of C18–C13–C17–O Dihedral in 1,2-Diketone **1** S7
9. Figure S6. Ortep Diagram of Polymorph **1A**. S8
10. Table S4. Geometrical Parameters Polymorph **1A**, Solid State S8
11. Figure S7. Ortep Diagram of Polymorph **1B**. S8
12. Table S5. Geometrical Parameters Polymorph **1B**, Solid State S8
13. Table S6. Crystal Data and Structure Refinements for Polymorphs **1A** and **1B** S9
14. Figure S8. Ortep Diagram of Compound **3**. S10
15. Table S7. Crystal Data and Structure Refinements for Compound **3** S10
16. Figure S9. PXRD pattern before irradiation of polymorph **1A** S11
17. Figure S10. PXRD pattern after irradiation of polymorph **1A** S11
18. Figure S11. PXRD pattern before irradiation of polymorph **1B** S12
19. Figure S12. PXRD pattern after irradiation of polymorph **1B** S12
20. Figure S13. PXDR pattern of polymorph **1A** after irradiation and simulated pattern of **3** S13
21. Figure S14. PXDR patterns of polymorphs **1A** and **1B**, before irradiation S14
22. ^{1}H and $^{13}C\{^{1}H\}$ NMR spectra of compound **1**. S15–S16
23. 1D and 2D NMR spectra of compound **2**. S17–S24
24. 1D and 2D NMR spectra of compound **3**. S25–S32
25. 1D and 2D NMR spectra of compound **4**. S33–S40

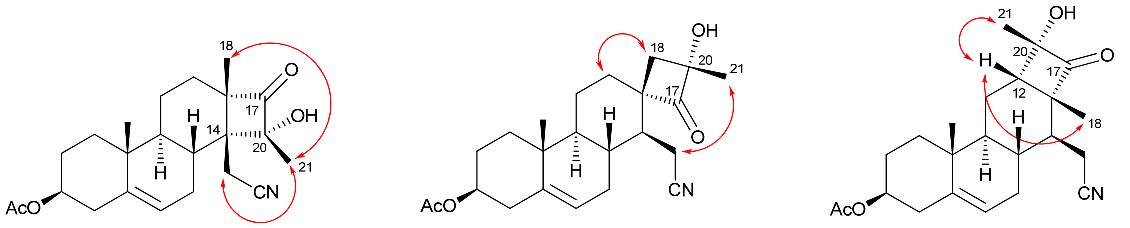

2 **3** **4**

Figure S1. Most Significant NOESY Correlations for Compounds **2–4**. All 2D spectra at 500 MHz, in $CDCl_3$.

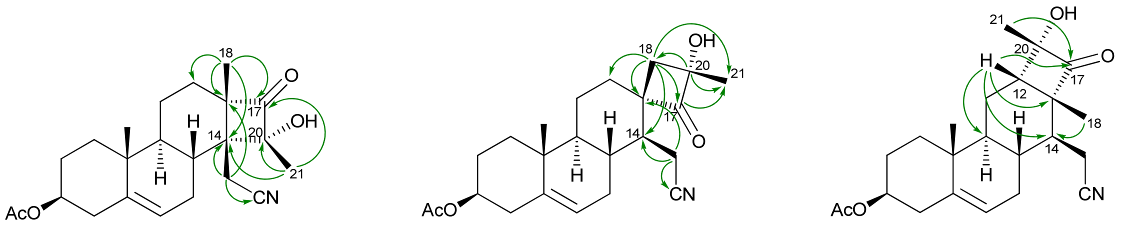

2 **3** **4**

Figure S2. Most Significant HMBC Correlations for Compounds **2–4**. All 2D spectra at 500 MHz, in $CDCl_3$, (H → C)

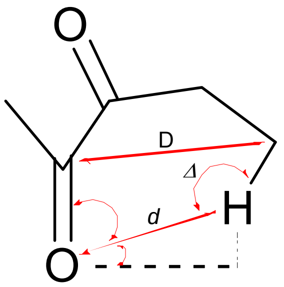

Figure S3. Definition of Geometrical Parameters D, *d*, *ω*, Δ, and *θ* for Intramolecular HAT.[1] The distance between the γ-hydrogen atom and the carbonyl oxygen atom (*d*), with the ideal value less than 2.4–2.7 Å; the distance between the reacting carbon atoms (D), with the ideal value less than 3.0 ± 0.09 Å; the C=O···H angle between the carbonyl group and the γ-hydrogen atom (Δ), with the ideal values in the range 90–120; the C–H···O angle formed by the γ-carbon, γ-hydrogen, and carbonyl oxygen (*θ*), with the ideal value 180°; the torsion angle describing the deviation of the γ-hydrogen from the plane of the carbonyl group (*ω*), with the ideal value 0°.

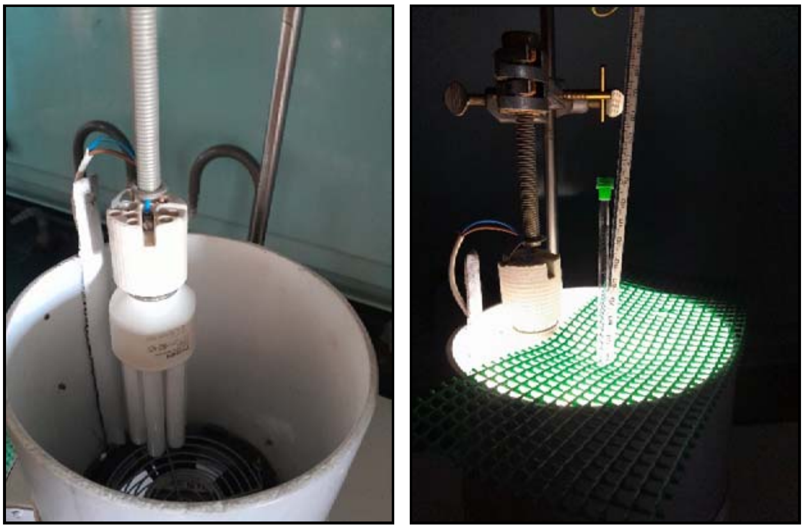

Figure S4. For the irradiation we used a homemade photoreactor, based in a white walls cylindrical plastic container, with a daylight lamp on the top and a fan on the bottom.

(1) (a) H. Ihmels, J. R. Scheffer, *Tetrahedron* **1999**, *55*, 885–907; (b) A. Natarajan, J. T. Mague, V. Ramamurthy, *J. Am. Chem. Soc.* **2005**, *127*, 3568–3576; (c) W. Xia, J. R. Scheffer, M. Botoshansky, M. Kaftory, *Org. Lett.* **2005**, *7*, 1315–1318.

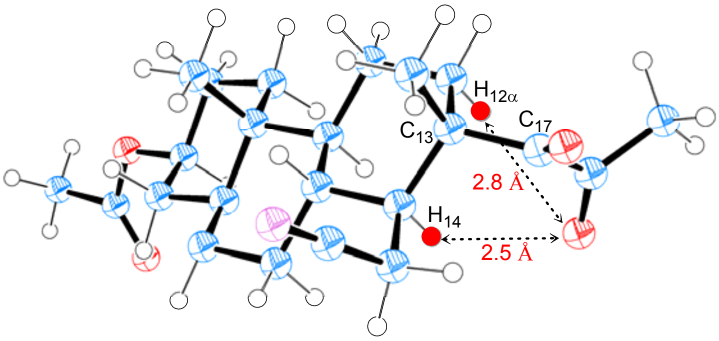

Table S1. Geometrical Parameters 1,2-Diketone **1**, Conformer **A**.[a]

Abstraction	*d* [Å]	D [Å]	*ω* [º]	Δ [º]	*θ* [º]
$H_{12\alpha}$	2.8	2.9	77.3	67.3	114.6
$H_{12\beta}$	3.9	2.9	73.2	43.7	54.5
H_{14}	2.5	3.2	27.6	96.8	121.1
H_{18}	4.8	3.8	28.3	35.2	64.0
Optimum value	2.4–2.7	3.0 ± 0.09	0	90–120	180

[a] Dihedral C_{18}–C_{13}–C_{17}–O = 20º. Relative energy = 0 kcal mol^{-1}. Potentially abstractable hydrogens are shown in red.

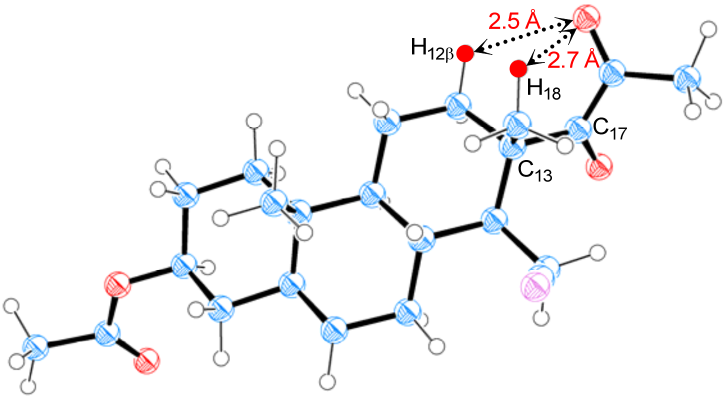

Table S2. Geometrical Parameters 1,2-Diketone **1**, Conformer **B**.[a]

Abstraction	*d* [Å]	D [Å]	*ω* [º]	Δ [º]	*θ* [º]
$H_{12\alpha}$	3.5	3.2	–4.2	80.5	64.2
$H_{12\beta}$	2.5	3.2	18.2	99.5	114.6
H_{14}	4.7	3.8	3.2	49.8	71.3
H_{18}	2.7	2.9	68.7	78.7	110.2
Optimum value	2.4–2.7	3.0 ± 0.09	0	90–120	180

[a] Dihedral C_{18}–C_{13}–C_{17}–O = 141º. Relative energy = 0.5 kcal mol^{-1}. Potentially abstractable hydrogens are shown in red.

Table S3. Coordinated scan calculation of C18–C13–C17–O dihedral in compound **1**[a]

Dihedral C18–C13–C17–O (°)	Relative Energy AMBER* (kcal mol^{-1})	Dihedral C18–C13–C17–O (°)	Relative Energy AMBER* (kcal mol^{-1})	Dihedral C18–C13–C17–O (°)	Relative Energy AMBER* (kcal mol^{-1})	Dihedral C18–C13–C17–O (°)	Relative Energy AMBER* (kcal mol^{-1})
–180	2.7294	–132	5.4171	–84	5.2298	–36	1.2595
–179	2.8156	–131	5.4066	–83	5.2123	–35	1.1865
–178	2.9048	–130	5.3929	–82	5.1879	–34	1.1151
–177	2.9959	–129	5.3760	–81	5.1576	–33	1.0459
–176	3.0893	–128	5.3582	–80	5.1220	–32	0.9802
–175	3.1834	–127	5.3432	–79	5.0778	–31	0.9171
–174	3.2790	–126	5.3276	–78	5.0252	–30	0.8543
–173	3.3779	–125	5.3118	–77	4.9675	–29	0.7958
–172	3.4777	–124	5.2927	–76	4.9023	–28	0.7394
–171	3.5792	–123	5.2759	–75	4.8318	–27	0.6848
–170	3.6830	–122	5.2592	–74	4.7555	–26	0.6333
–169	3.7893	–121	5.2429	–73	4.6712	–25	0.5853
–168	3.8957	–120	5.2289	–72	4.5858	–24	0.5395
–167	4.0040	–119	5.2164	–71	4.4965	–23	0.4954
–166	4.1138	–118	5.2037	–70	4.4027	–22	0.4536
–165	4.2231	–117	5.1902	–69	4.3086	–21	0.4144
–164	4.3324	–116	5.1786	–68	4.2094	–20	0.3777
–163	4.4449	–115	5.1678	–67	4.1060	–19	0.3440
–162	4.5572	–114	5.1597	–66	4.0053	–18	0.3107
–161	4.6687	–113	5.1521	–65	3.9000	–17	0.2819
–160	4.7793	–112	5.1469	–64	3.7962	–16	0.2556
–159	4.8846	–111	5.1429	–63	3.6910	–15	0.2302
–158	4.9857	–110	5.1391	–62	3.5871	–14	0.2066
–157	4.8816	–109	5.1372	–61	3.4833	–13	0.1855
–156	4.9325	–108	5.1358	–60	3.3804	–12	0.1676
–155	4.9820	–107	5.1371	–59	3.2770	–11	0.1506
–154	5.0275	–106	5.1396	–58	3.1749	–10	0.1352
–153	5.0702	–105	5.1420	–57	3.0747	–9	0.1226
–152	5.1087	–104	5.1468	–56	2.9780	–8	0.1112
–151	5.1493	–103	5.1511	–55	2.8807	–7	0.1019
–150	5.1865	–102	5.1586	–54	2.7860	–6	0.0934
–149	5.2192	–101	5.1635	–53	2.6936	–5	0.0863
–148	5.2513	–100	5.1699	–52	2.6011	–4	0.0815
–147	5.2791	–99	5.1780	–51	2.5112	–3	0.0767
–146	5.3060	–98	5.1856	–50	2.4222	–2	0.0734
–145	5.3313	–97	5.1950	–49	2.3357	–1	0.0702
–144	5.3521	–96	5.2017	–48	2.2506	0	0.0679
–143	5.3704	–95	5.2116	–47	2.1661	1	0.0672
–142	5.3898	–94	5.2229	–46	2.0814	2	0.0656
–141	5.4045	–93	5.2304	–45	1.9976	3	0.0643
–140	5.4136	–92	5.2377	–44	1.9112	4	0.0620
–139	5.4223	–91	5.2430	–43	1.8252	5	0.0607
–138	5.4312	–90	5.2504	–42	1.7415	6	0.0579
–137	5.4361	–89	5.2551	–41	1.6583	7	0.0551
–136	5.4378	–88	5.2540	–40	1.5749	8	0.0525
–135	5.4361	–87	5.2539	–39	1.4933	9	0.0496
–134	5.4342	–86	5.2500	–38	1.4140	10	0.0475
–133	5.4268	–85	5.2414	–37	1.3356	11	0.0429

Dihedral C18–C13–C17–O (°)	Relative Energy AMBER* (kcal mol⁻¹)	Dihedral C18–C13–C17–O (°)	Relative Energy AMBER* (kcal mol⁻¹)	Dihedral C18–C13–C17–O (°)	Relative Energy AMBER* (kcal mol⁻¹l)	Dihedral C18–C13–C17–O (°)	Relative Energy AMBER* (kcal mol⁻¹)
12	0.0376	55	1.8765	98	2.2830	**141**	**0.5560**
13	0.0324	56	1.9612	99	2.2248	142	0.5586
14	0.0267	57	2.0451	100	2.1658	143	0.5644
15	0.0202	58	2.1247	101	2.1055	144	0.5759
16	0.0138	59	2.2079	102	2.0472	145	0.5895
17	0.0085	60	2.2851	103	1.9862	146	0.6073
18	0.0042	61	2.3637	104	1.9287	147	0.6295
19	0.0014	62	2.4376	105	1.8715	148	0.6550
**20**	**0.0000**	63	2.5069	106	1.8137	149	0.6851
21	0.0016	64	2.5731	107	1.7571	150	0.7183
22	0.0037	65	2.6386	108	1.7012	151	0.7558
23	0.0097	66	2.6975	109	1.6465	152	0.7962
24	0.0170	67	2.7558	110	1.5945	153	0.8395
25	0.0307	68	2.8069	111	1.5421	154	0.8875
26	0.0475	69	2.8535	112	1.4938	155	0.9371
27	0.0686	70	2.8919	113	1.4406	156	0.9901
28	0.0928	71	2.9282	114	1.3947	157	1.0467
29	0.1193	72	2.9593	115	1.3463	158	1.1047
30	0.1486	73	2.9833	116	1.3013	159	1.1636
31	0.1838	74	3.0013	117	1.2570	160	1.2256
32	0.2215	75	3.0167	118	1.2146	161	1.2905
33	0.2653	76	3.0291	119	1.1763	162	1.3550
34	0.3100	77	3.0362	120	1.1341	163	1.4215
35	0.3620	78	3.0400	121	1.0945	164	1.4909
36	0.4168	79	3.0377	122	1.0554	165	1.5613
37	0.4738	80	3.0312	123	1.0176	166	1.6330
38	0.5337	81	3.0202	124	0.9800	167	1.7026
39	0.5960	82	3.0060	125	0.9457	168	1.7773
40	0.6629	83	2.9841	126	0.9110	169	1.8488
41	0.7312	84	2.9590	127	0.8780	170	1.9219
42	0.8025	85	2.9307	128	0.8446	171	1.9983
43	0.8781	86	2.8976	129	0.8099	172	2.0742
44	0.9532	87	2.8601	130	0.7771	173	2.1504
45	1.0335	88	2.8185	131	0.7460	174	2.2283
46	1.1155	89	2.7735	132	0.7146	175	2.3076
47	1.1975	90	2.7287	133	0.6858	176	2.3875
48	1.2783	91	2.6762	134	0.6581	177	2.4696
49	1.3624	92	2.6252	135	0.6315	178	2.5519
50	1.4475	93	2.5710	136	0.6087	179	2.6361
51	1.5333	94	2.5156	137	0.5906	180	2.7222
52	1.6183	95	2.4597	138	0.5755		
53	1.7045	96	2.4000	139	0.5645		
54	1.7912	97	2.3429	140	0.5580		

[a] Molecular mechanics calculations performed with AMBER* force field as implemented in MacroModel v 9.7 with the GB/SA solvent model for $CHCl_3$, Schrödinger, LLC, New York. The minimum for each conformational isomer was calculated performing a coordinated scan calculation of C18–C13–C17–O dihedral in increments of 1°. The dicarbonyl system deviates appreciably from *trans* coplanarity. Intercarbonyl dihedral angle of 134.5° as measured in the X-ray structure was used for the study.

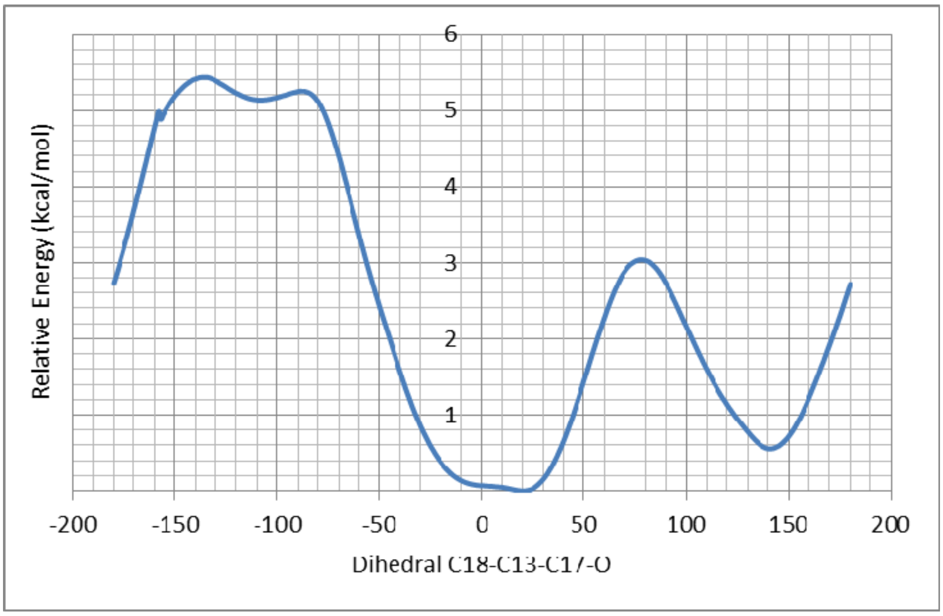

Figure S5. Coordinated scan of C18–C13–C17–O dihedral in compound **1**

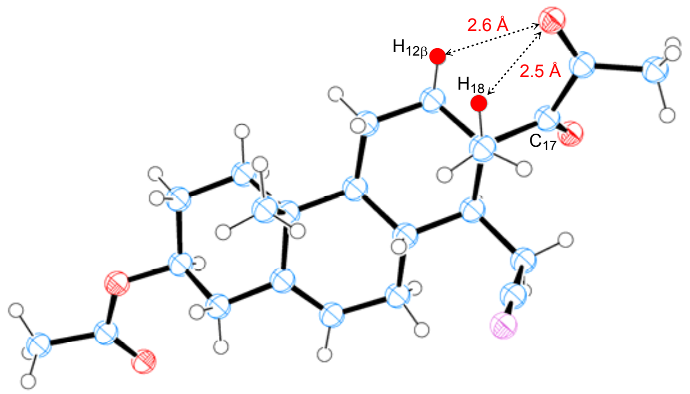

Figure S6. Ortep Diagram of Polymorph **1A** with 50% Probability Ellipsoid.

Table S4. Geometrical Parameters of Polymorph **1A**, Solid State, X-Ray Crystallography.[a]

Abstraction	*d* [Å]	D [Å]	*ω* [°]	Δ [°]	*θ* [°]
$H_{12\alpha}$	3.7	3.3	–12.5	77.6	57.6
$H_{12\beta}$	2.6	3.3	–1.6	97.4	120.5
H_{14}	4.8	3.9	3.5	51.5	68.1
H_{18}	2.5	2.9	54.1	88.1	115.9
Optimum value	2.4–2.7	3.0 ± 0.09	0	90–120	180

[a] Dihedral C_{18}–C_{13}–C_{17}–O = 148.17(13)°. Potentially abstractable hydrogens are shown in red.

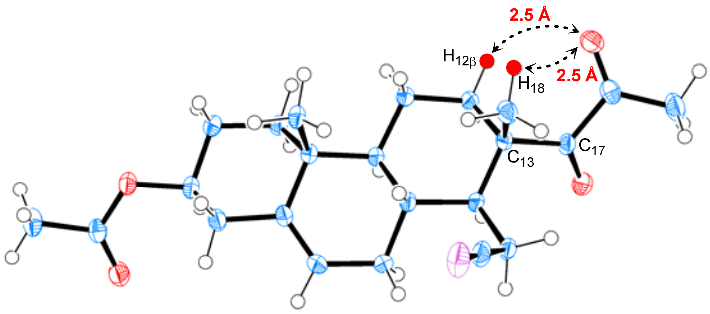

Figure S7. Ortep Diagram of Polymorph **1B** with 50% Probability Ellipsoid.

Table S5. Geometrical Parameters of Polymorph **1B**, Solid State, X-Ray Crystallography.[a]

Abstraction	*d* [Å]	D [Å]	*ω* [°]	Δ [°]	*θ* [°]
$H_{12\alpha}$	3.5	3.3	–18.6	79.8	58.5
$H_{12\beta}$	2.5	3.3	–7.7	99.7	122.2
H_{14}	4.7	4.0	–0.6	56.5	70.0
H_{18}	2.5	3.0	49.7	91.1	114.1
Optimum value	2.4–2.7	3.0 ± 0.09	0	90–120	180

[a] Dihedral C_{18}–C_{13}–C_{17}–O = 142.3(2)°. Potentially abstractable hydrogens are shown in red.

**Table S6.** Crystal Data and Structure Refinements for Polymorphs **1A** and **1B**.		
Polymorph	**1A**	**1B**
Empirical formula	$C_{23}H_{31}NO_4$	$C_{23}H_{31}NO_4$
Formula weight	385.49	385.49
Temperature (K)	123(2)	123(2)
Wavelength (Å)	1.54180	1.54180
Crystal system	Monoclinic	Monoclinic
Space group	$P2_1$	$P2_1$
Unit cell dimensions		
a (Å)	9.5769(1)	7.6474(3)
b (Å)	9.8814(1)	15.4448(5)
c (Å)	10.6257(1)	9.2459(3)
β (°)	93.098(1)	111.210(4)
Volume (Å^3)	1004.074(17)	1018.08(7)
Z	2	2
Density (calculated) (Mg/m^3)	1.275	1.257
Absorption coefficient (mm^{-1})	0.693	0.683
F(000)	416	416
Crystal size (mm^3)	0.34 × 0.28 × 0.10	0.30 × 0.25 × 0.05
Theta range for data collection (°)	4.17 to 71.83.	5.129 to 72.767.
Index ranges	–11<=h<=11, –12<=k<=11, –13<=l<=13	–9<=h<=6, –18<=k<=18, –11<=l<=11
Reflections collected	13014	4100
Independent reflections	3655 [R(int) = 0.0133]	3103 [R(int) = 0.0136]
Completeness to theta = 70.00°	99.7 %	99.7 %
Absorption correction	Semi-empirical from equivalents	Semi-empirical from equivalents
Max. and min. transmission	1.00000 and 0.68513	1.00000 and 0.34559
Refinement method	Full-matrix least-squares on F^2	Full-matrix least-squares on F^2
Data / restraints / parameters	3655 / 1 / 257	3103 / 1 / 257
Goodness-of-fit on F^2	1.045	1.044
Final *R* indices [I>2σ(I)]	R_1 = 0.0316, wR_2 = 0.0850	R_1 = 0.0382, wR_2 = 0.1028
R indices (all data)	R_1 = 0.0319, wR_2 = 0.0853	R_1 = 0.0384, wR_2 = 0.1034
Absolute structure parameter	–0.01(13)	0.17(11)
Largest diff. peak and hole	0.260 and -0.153 e.Å^{-3}	0.300 and -0.227 e.Å^{-3}

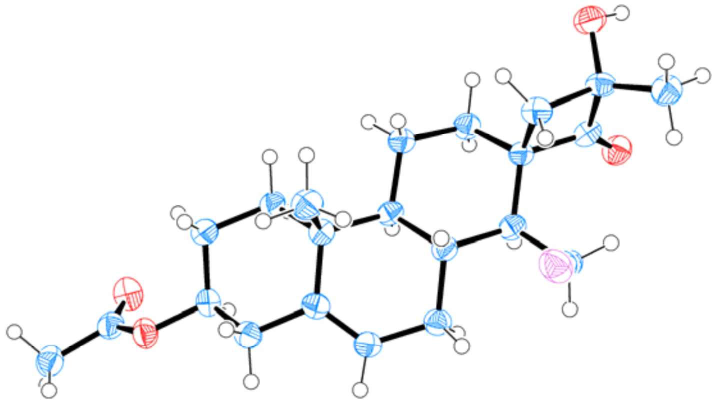

Figure S8. Ortep Diagram of Compound **3** with 50% Probability Ellipsoid.

**Table S7.** Crystal data and structure refinement for compound **3**.	
Empirical formula	$C_{23}H_{31}NO_4$
Formula weight	385.49
Temperature	123(2) K
Wavelength	1.54180 Å
Crystal system	Orthorhombic
Space group	$P2_12_12_1$
Unit cell dimensions	
a (Å)	7.7874(13)
b (Å)	9.0718(19)
c (Å)	28.882(6)
Volume (Å^3)	2040.4(7)
Z	4
Density (calculated) (Mg/m^3)	1.255
Absorption coefficient (mm^{-1})	0.682
F(000)	832
Crystal size (mm^3)	0.28 × 0.20 × 0.05
Theta range for data collection	5.11 to 67.95°.
Index ranges	–8<=h<=9, –10<=k<=10, –34<=l<=34
Reflections collected	10623
Independent reflections	3627 [R(int) = 0.0645]
Completeness to theta = 67.95°	99.0 %
Absorption correction	Semi-empirical from equivalents
Max. and min. transmission	1.00000 and 0.88181
Refinement method	Full-matrix least-squares on F^2
Data / restraints / parameters	3627 / 0 / 260
Goodness-of-fit on F^2	0.865
Final *R* indices [I>2σ(I)]	R_1 = 0.0492, wR_2 = 0.0856
R indices (all data)	R_1 = 0.1113, wR_2 = 0.1073
Absolute structure parameter	0.0(4)
Largest diff. peak and hole	0.225 and -0.258 e.Å^{-3}

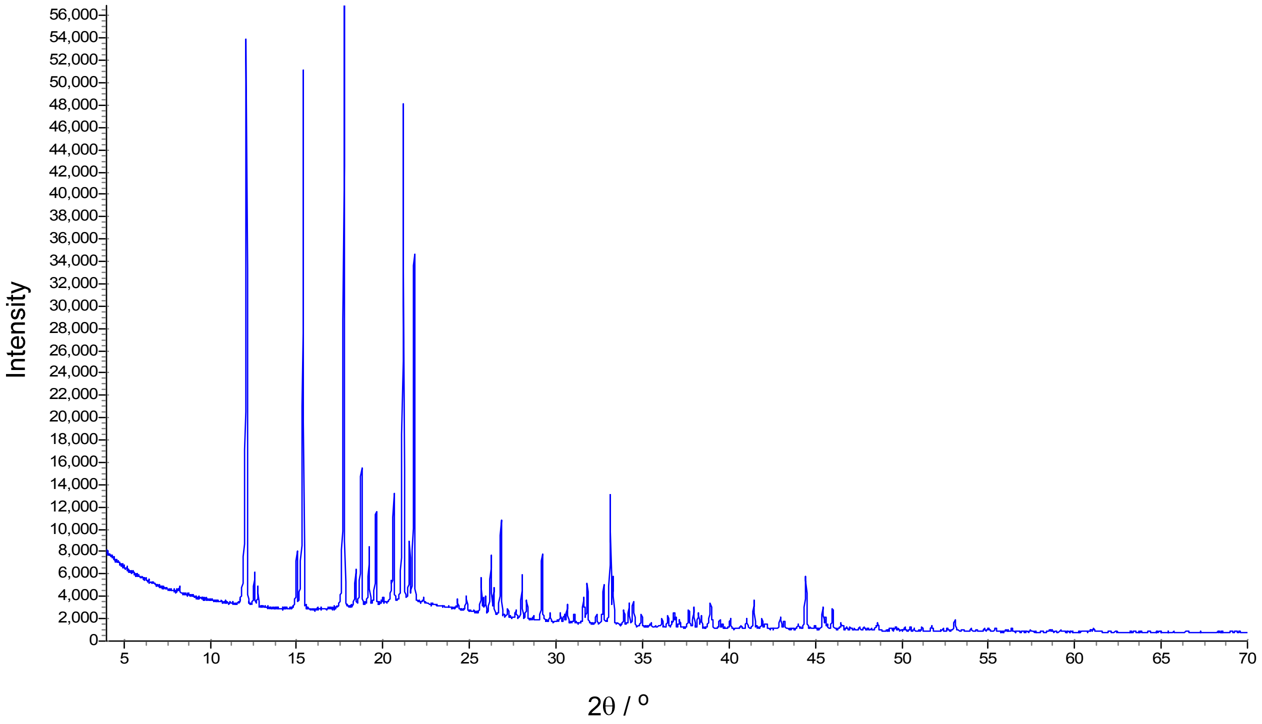

Figure S9. Powder X-ray diffraction pattern before irradiation of polymorph **1A**

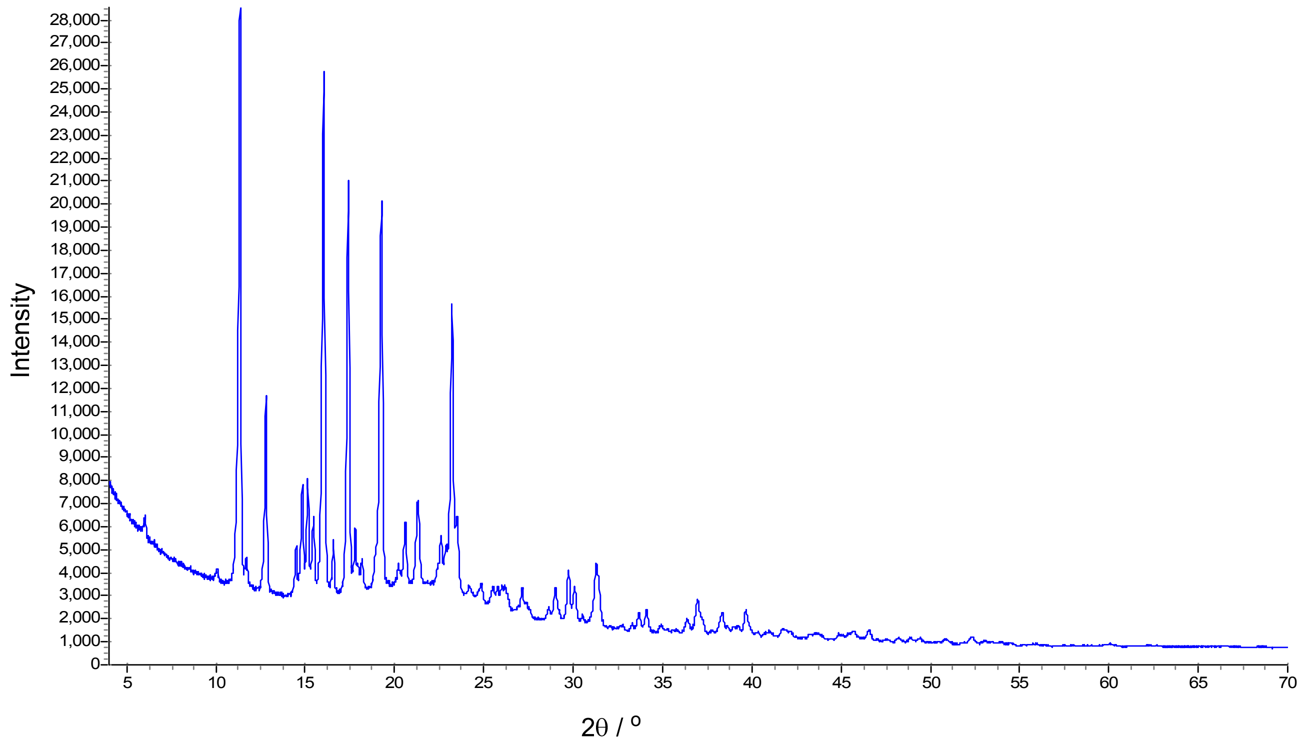

Figure S10. Powder X-ray diffraction pattern after irradiation of polymorph **1A**

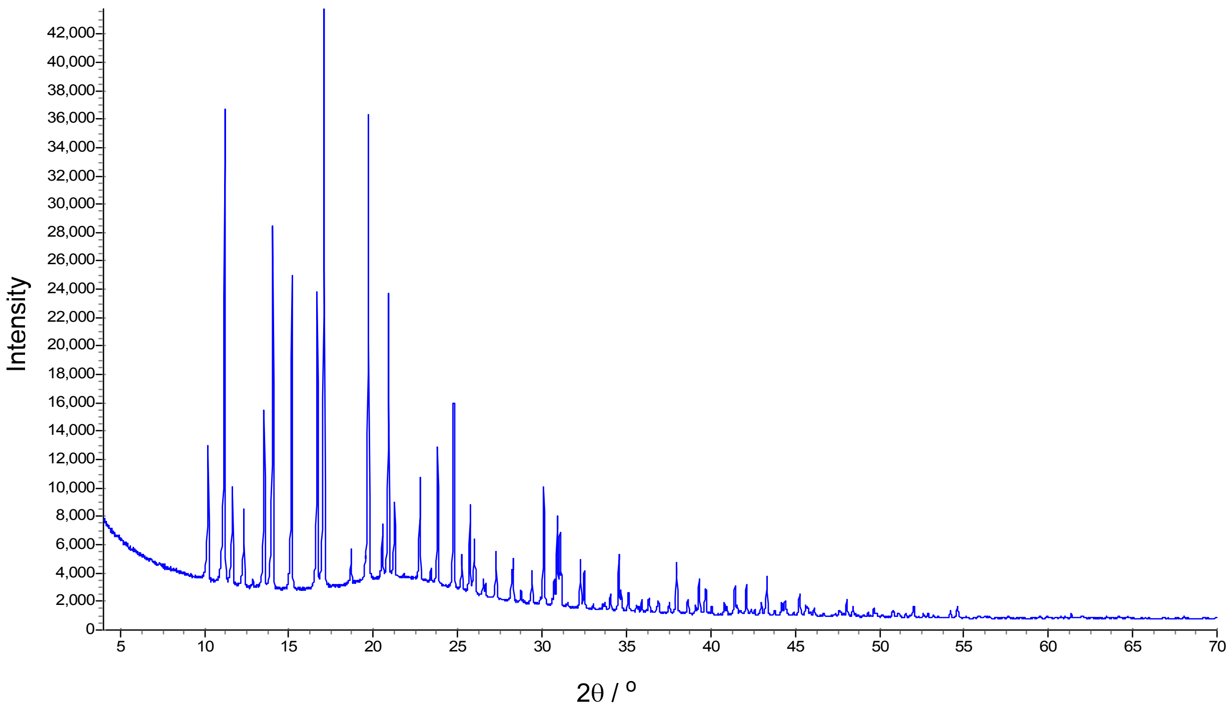

Figure S11. Powder X-ray diffraction pattern before irradiation of polymorph **1B**

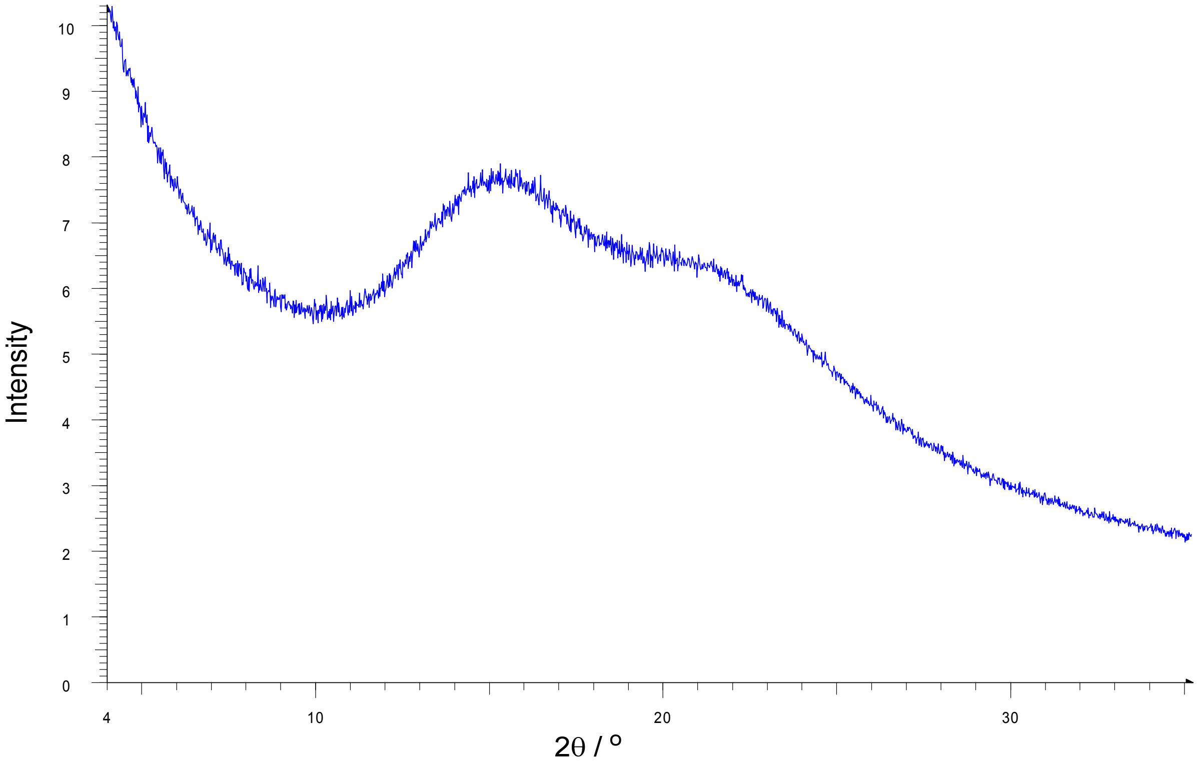

Figure S12. Powder X-ray diffraction pattern after irradiation of polymorph **1B**, run truncated at 35 2θ / °, as no Bragg diffraction evident

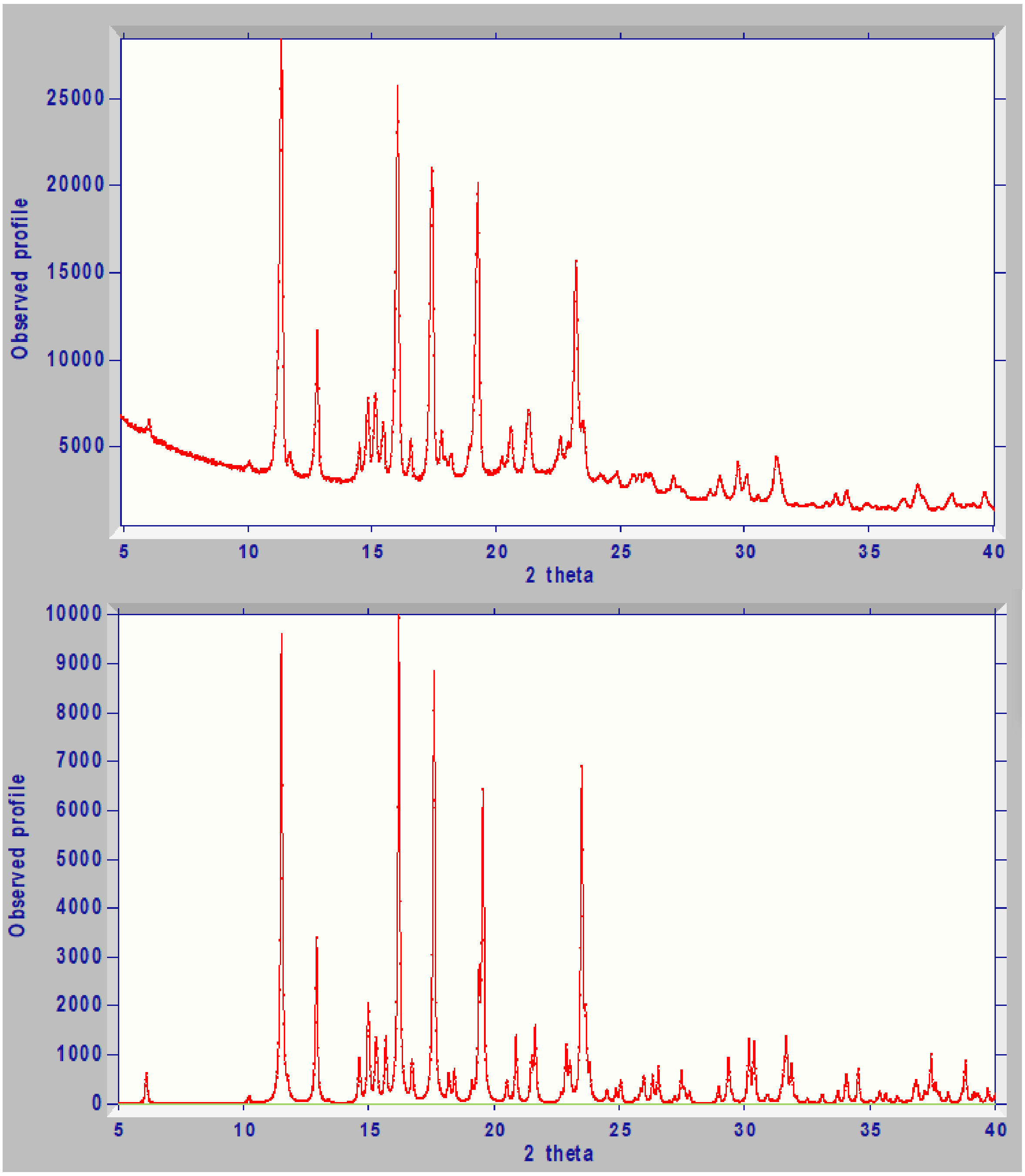

Figure S13. Powder X-ray diffraction pattern of polymorph **1A** at room temperature (upper) after irradiation and simulated powder X-ray diffraction pattern of **3** at 123 K (lower) in the range 5-40° 2θ, showing excellent agreement. Small changes in peak positions are attributable to the temperature difference between the two datasets.

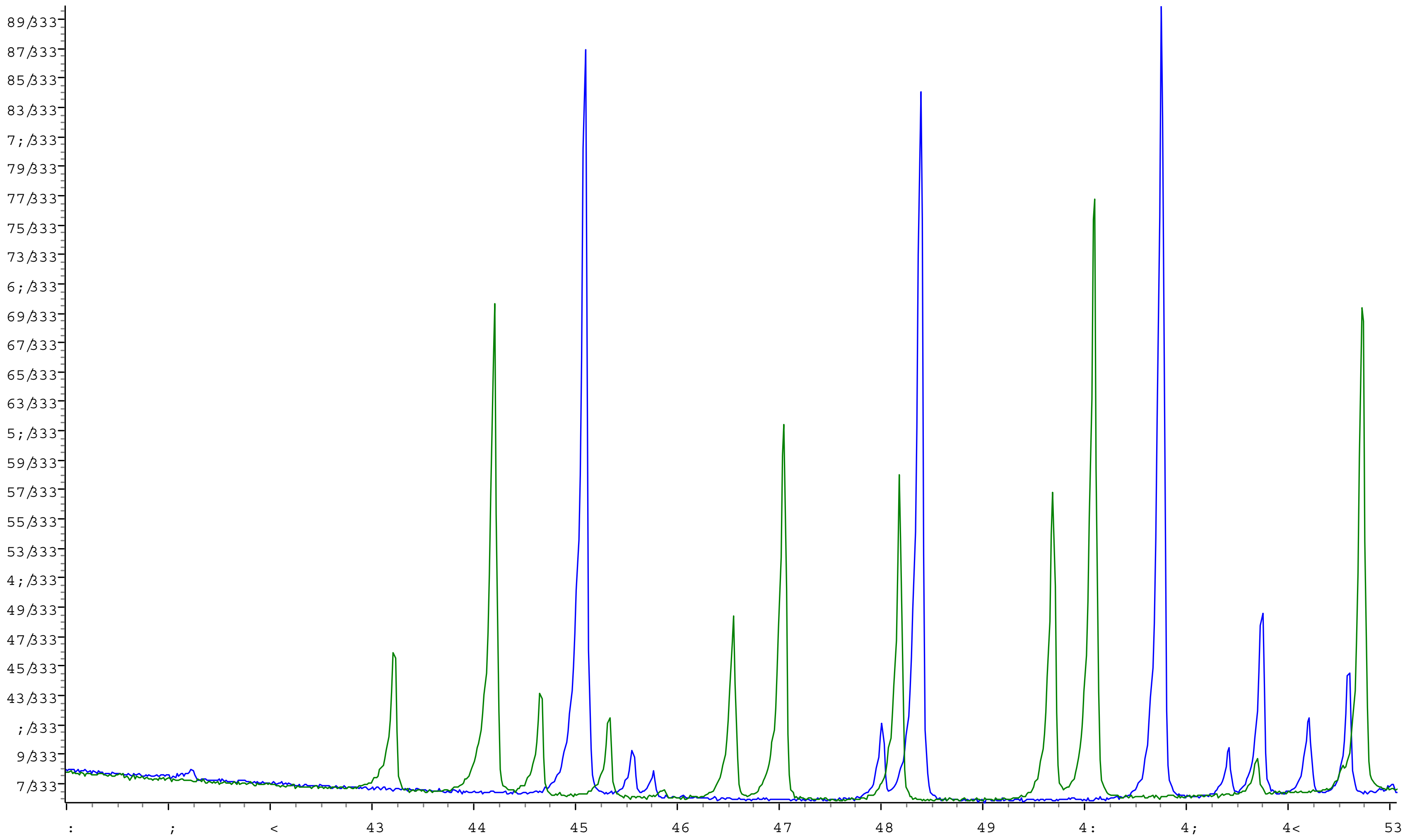

Figure S14. Powder X-ray diffraction patterns of polymorphs **1A** (blue) and **1B** (green) in the range 7-20° 2θ, before irradiation

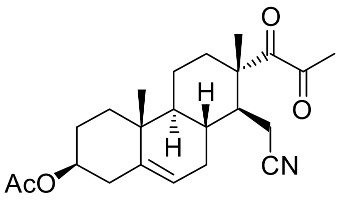

1

^{1}H NMR (400 MHz, $CDCl_3$)

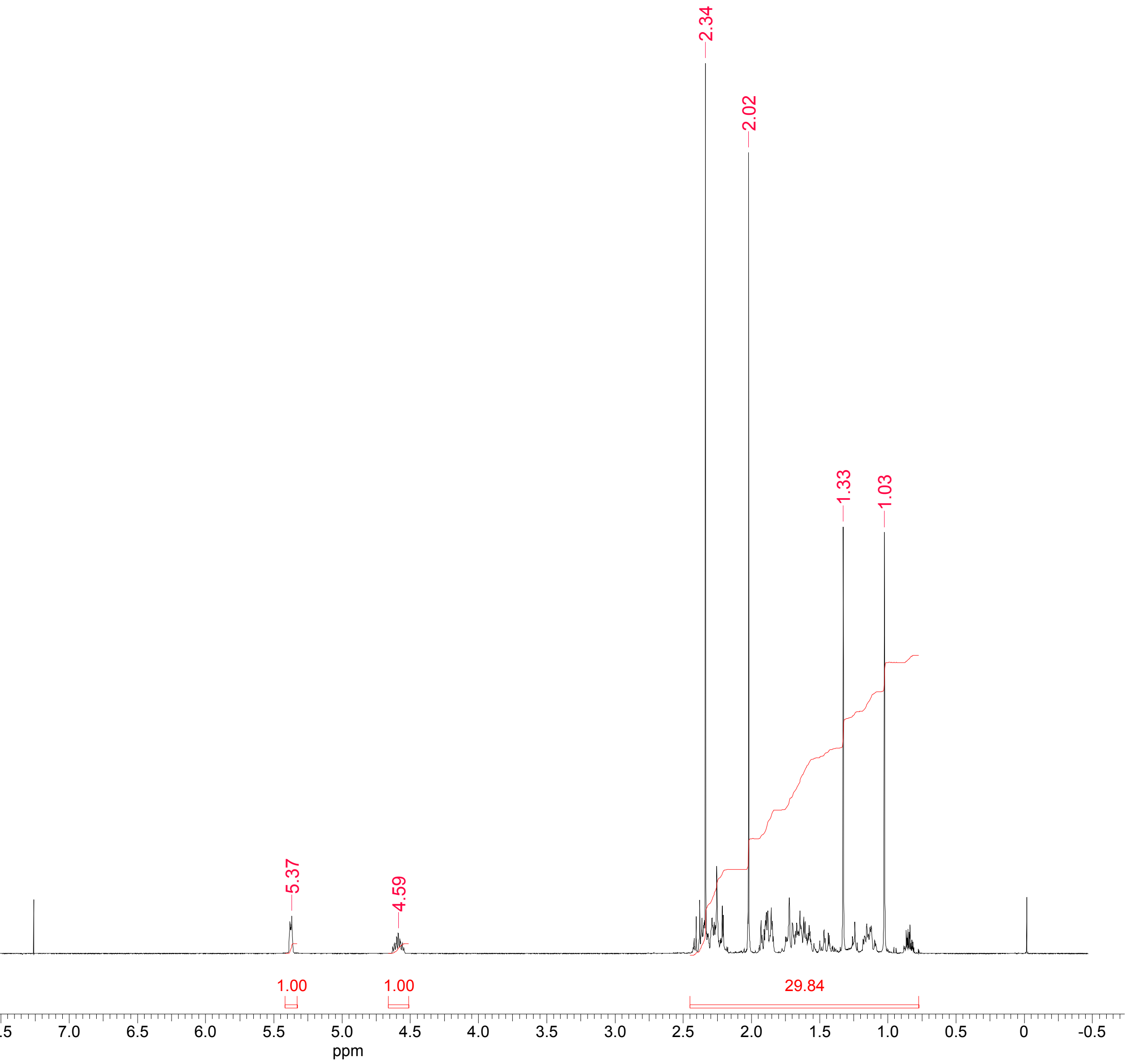

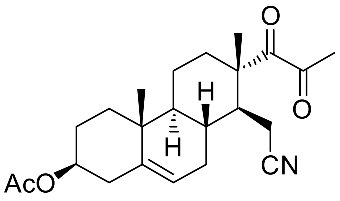

1

$^{13}C\{^1H\}$ NMR (100.4 MHz, $CDCl_3$)

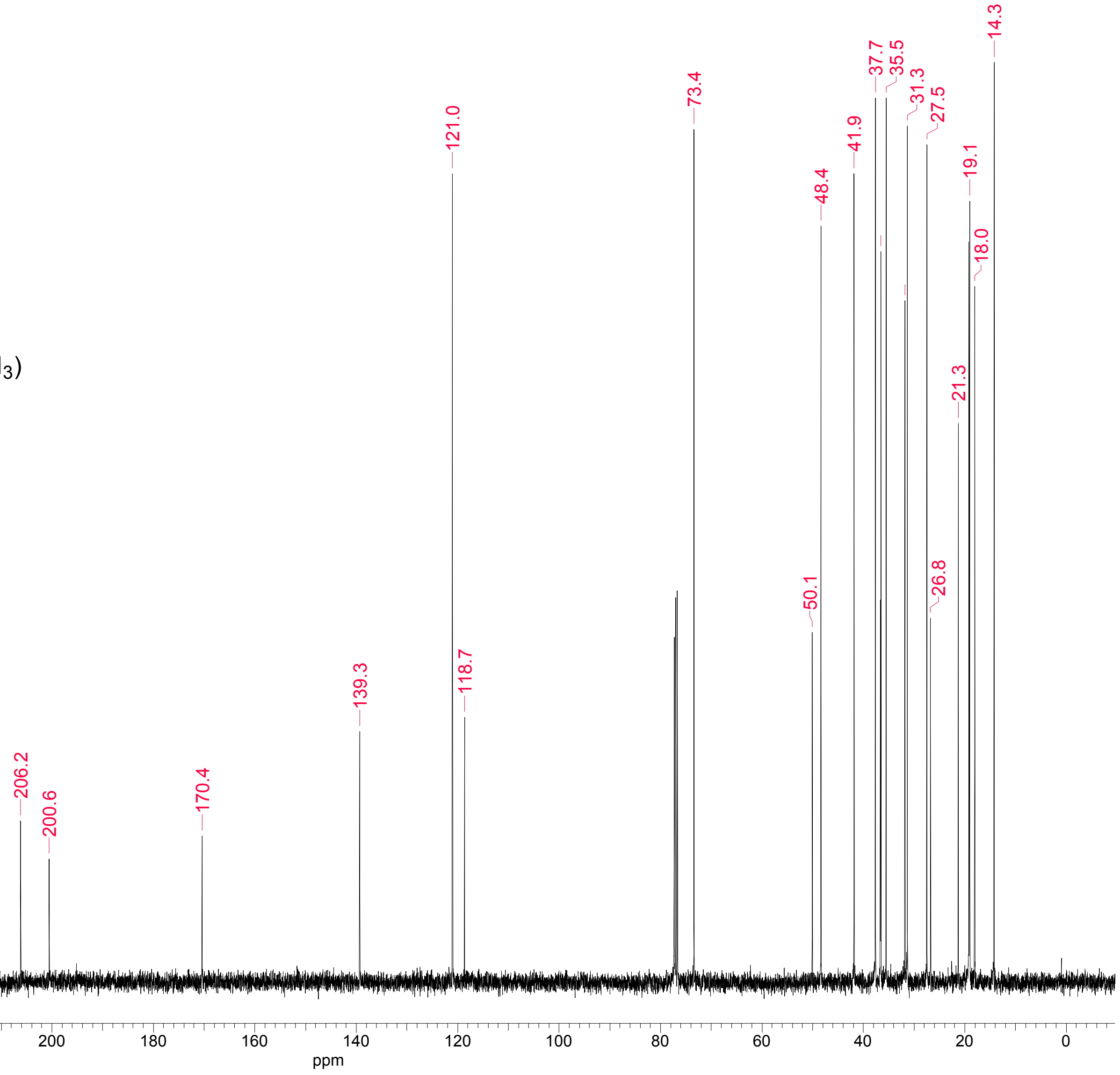

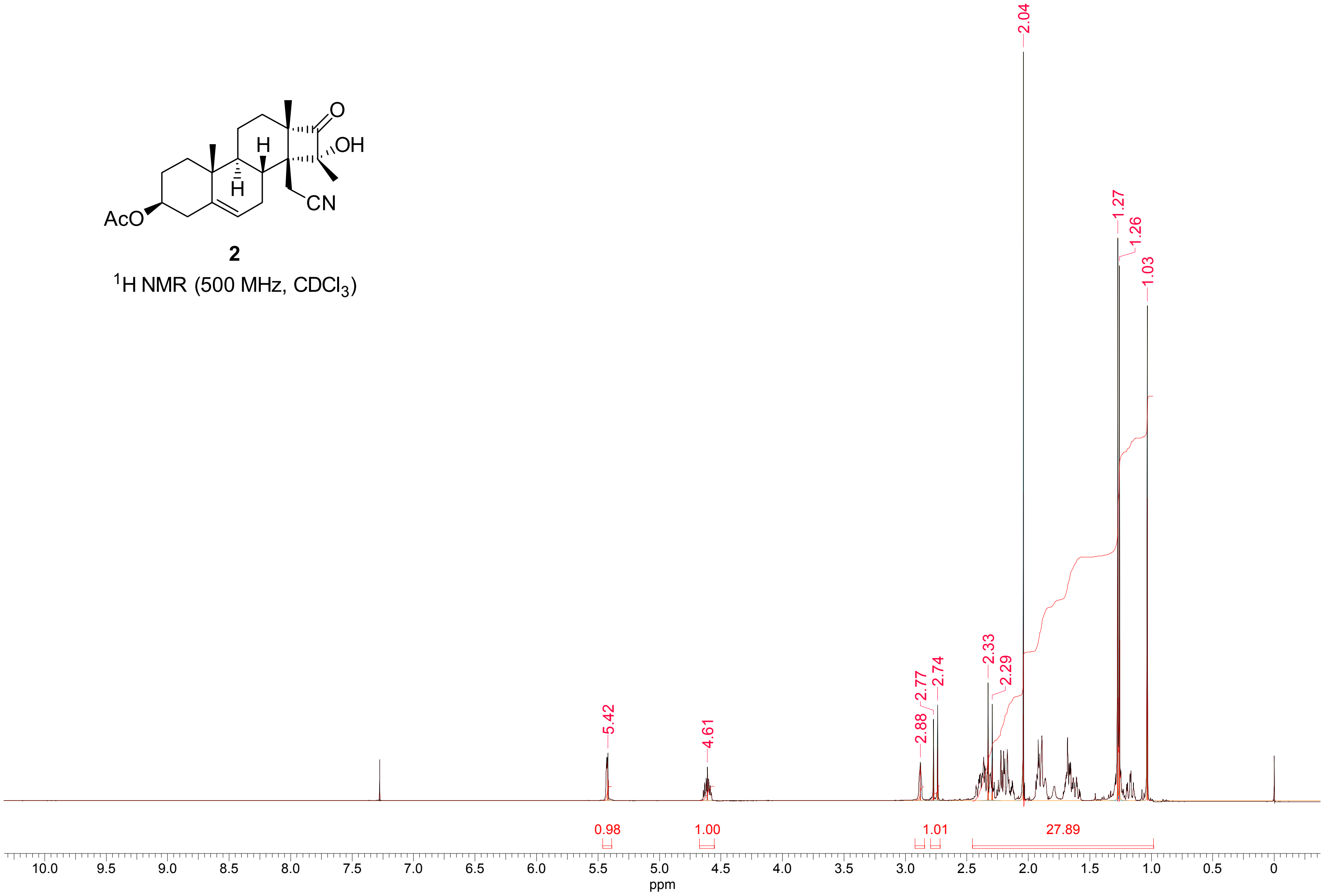

2

^1H NMR (500 MHz, $CDCl_3$)

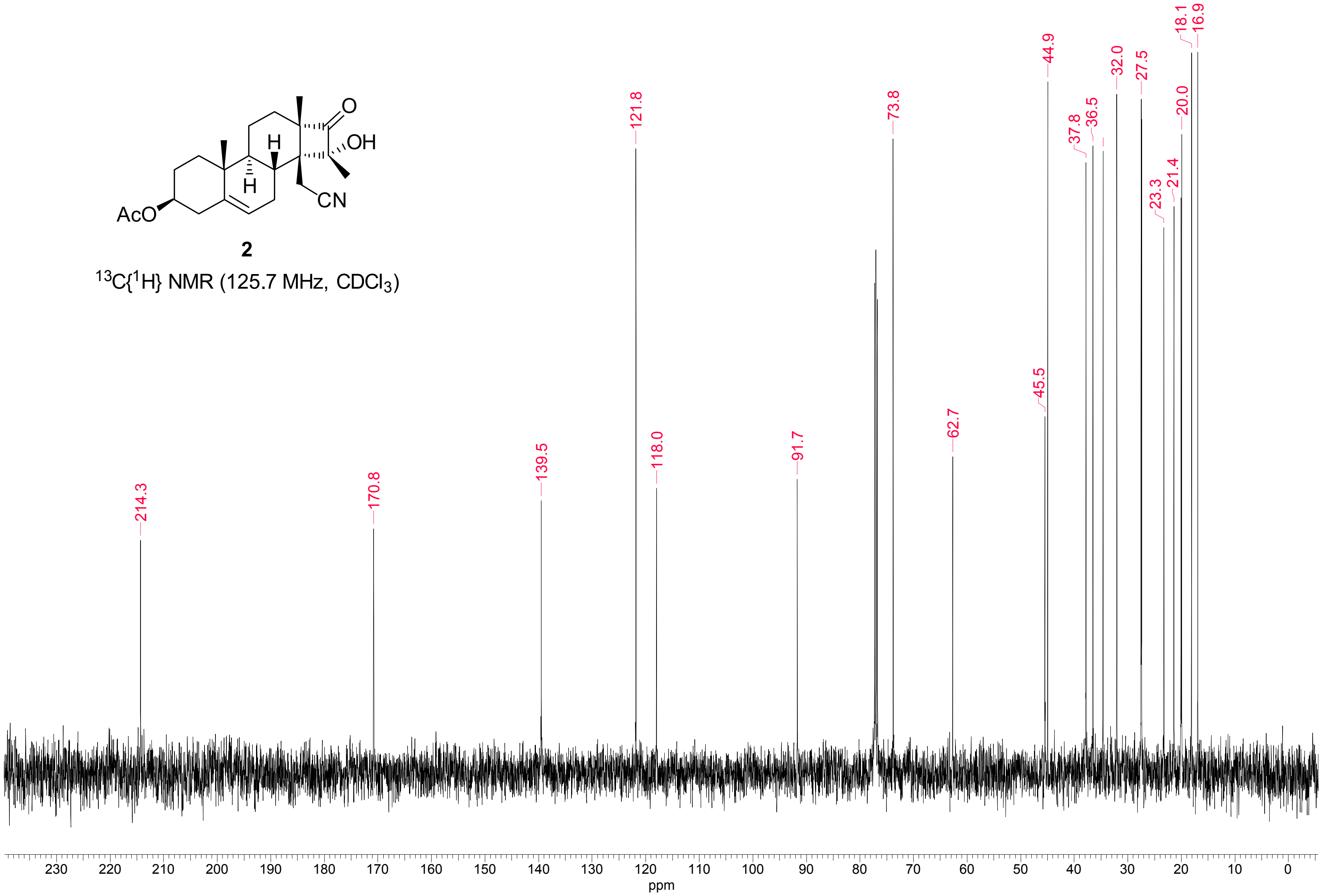

2

$^{13}C\{^{1}H\}$ NMR (125.7 MHz, $CDCl_3$)

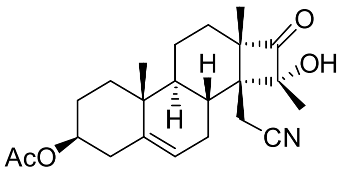

2

DEPT90 (125.7 MHz, $CDCl_3$)

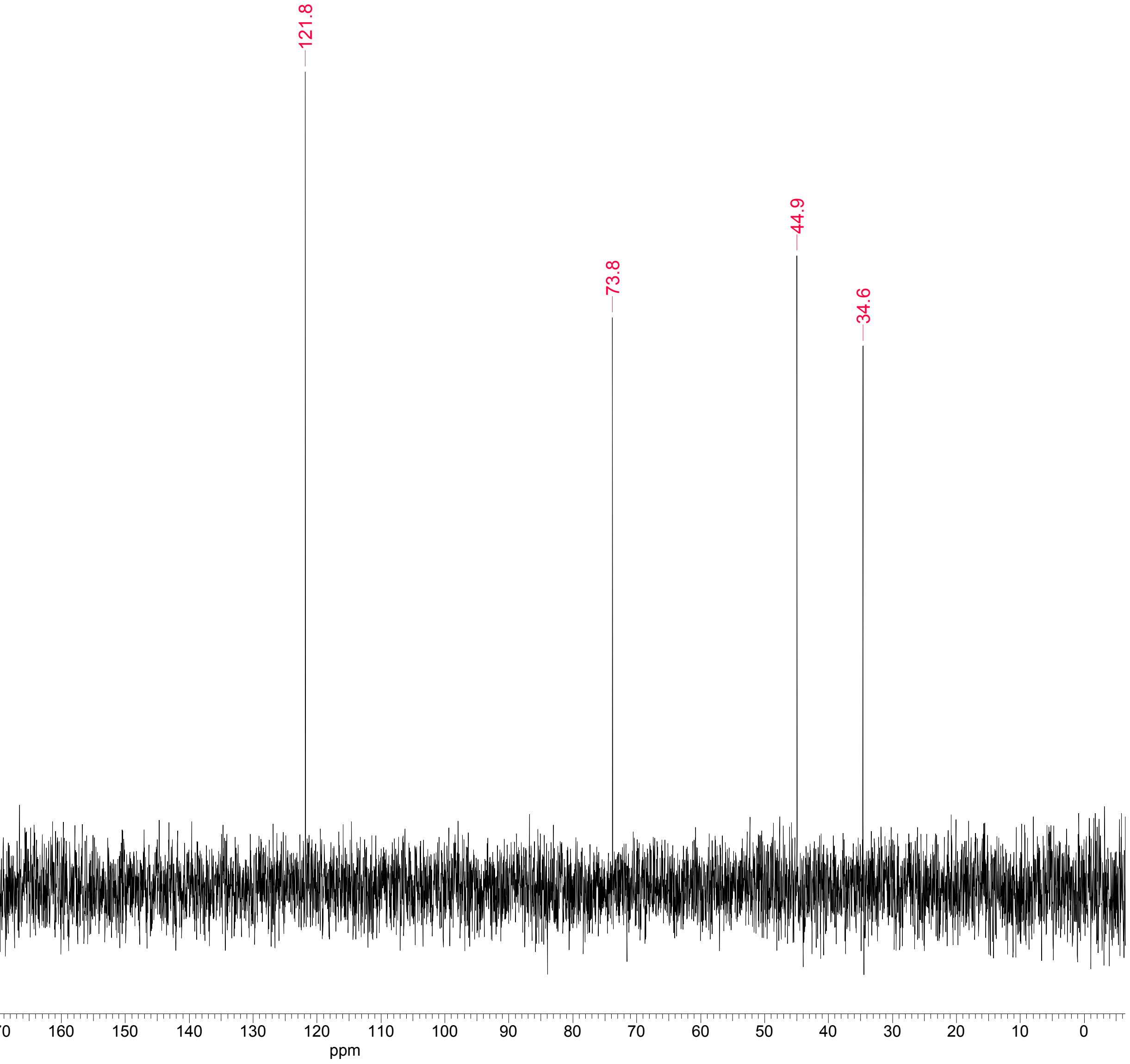

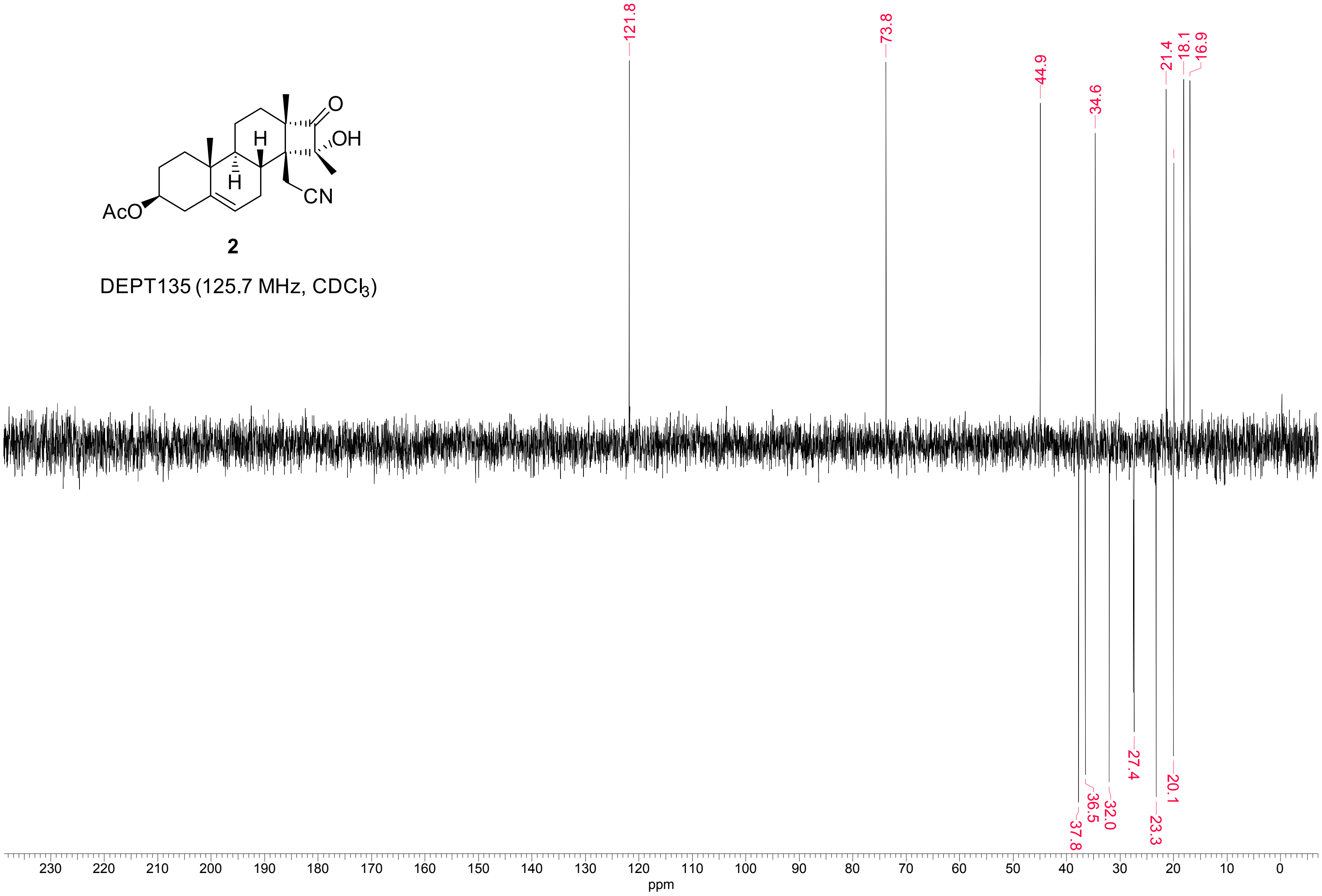

2

DEPT135 (125.7 MHz, $CDCl_3$)

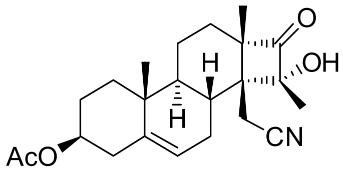

2

COSY (500 MHz, $CDCl_3$)

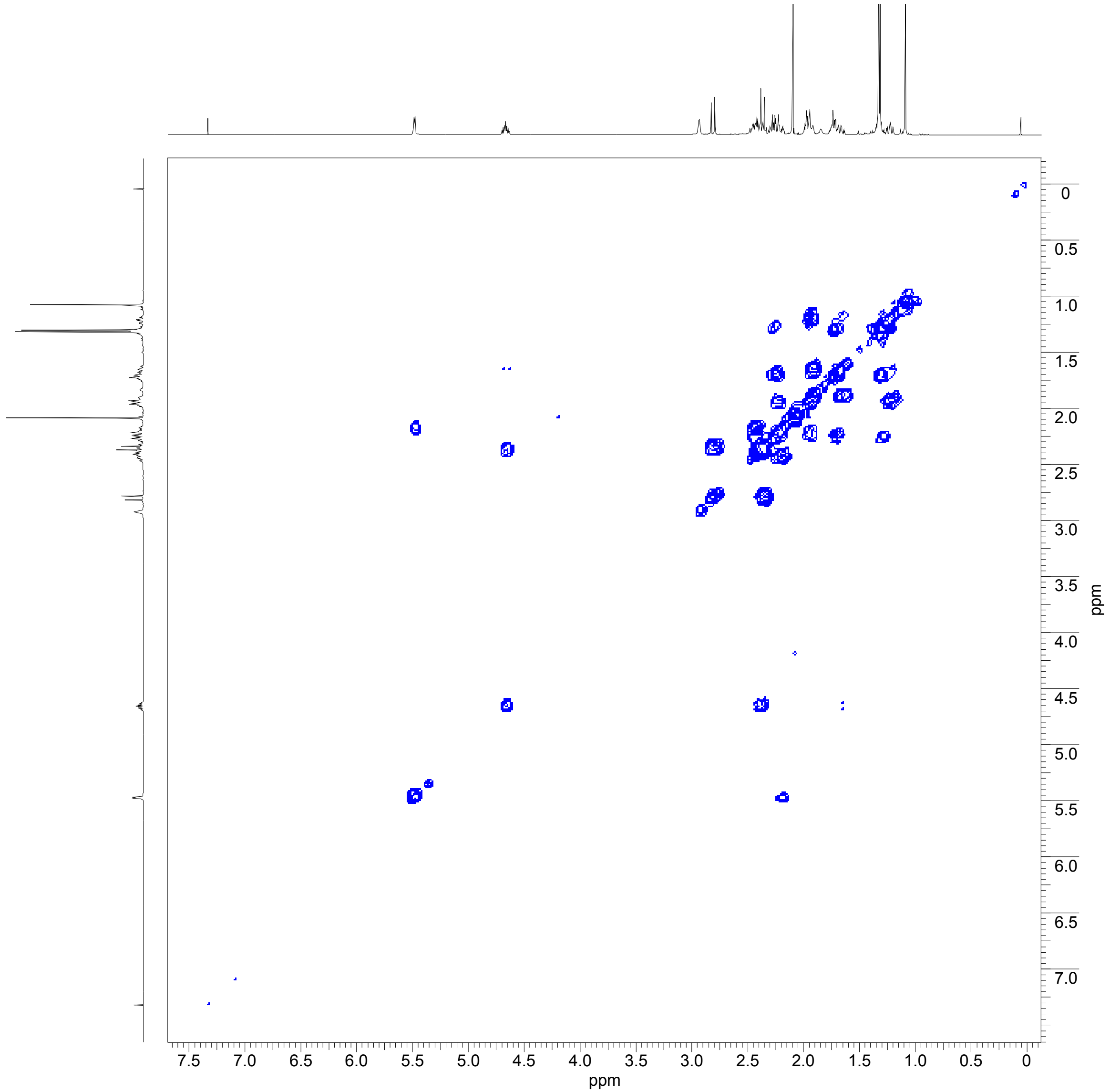

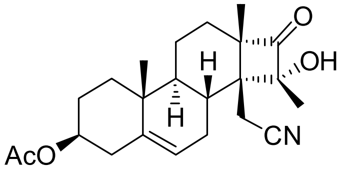

2

NOESY (500 MHz, $CDCl_3$)

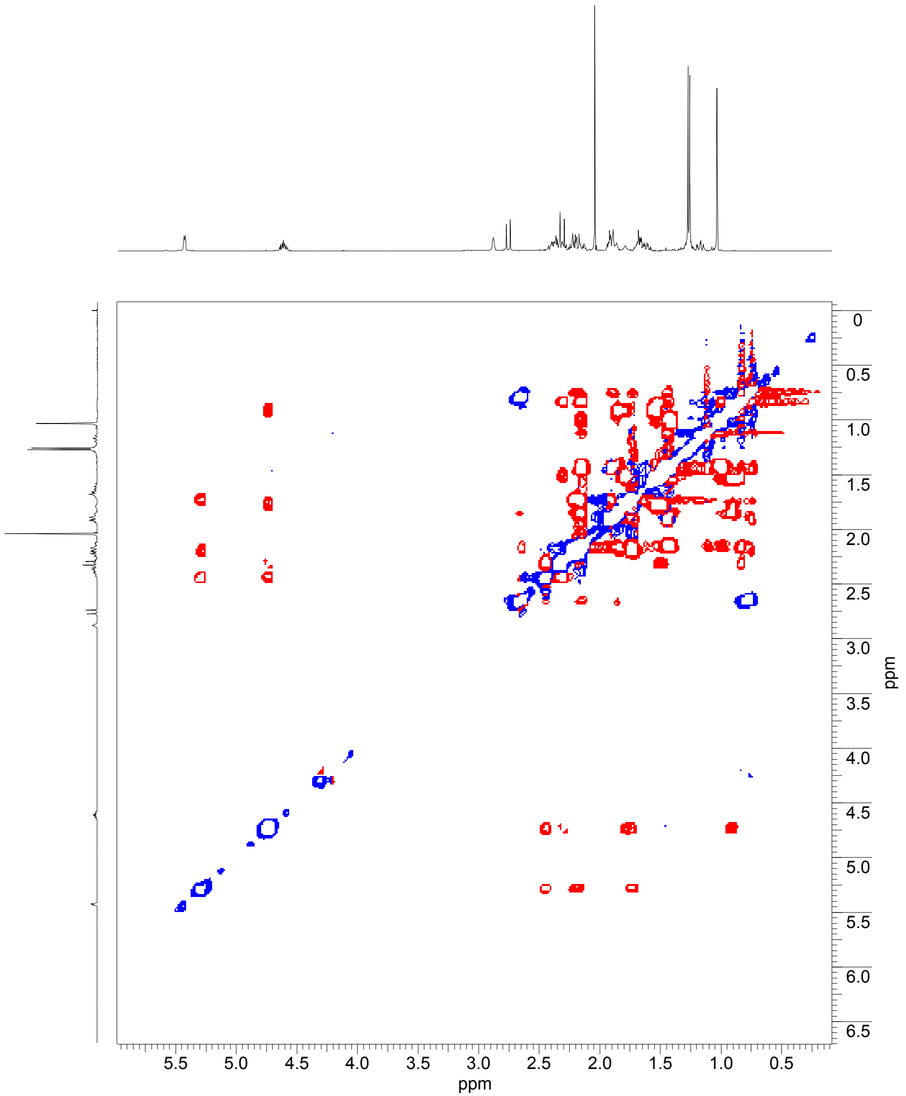

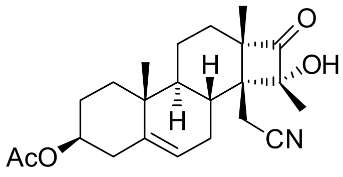

2

HSQC ($CDCl_3$)

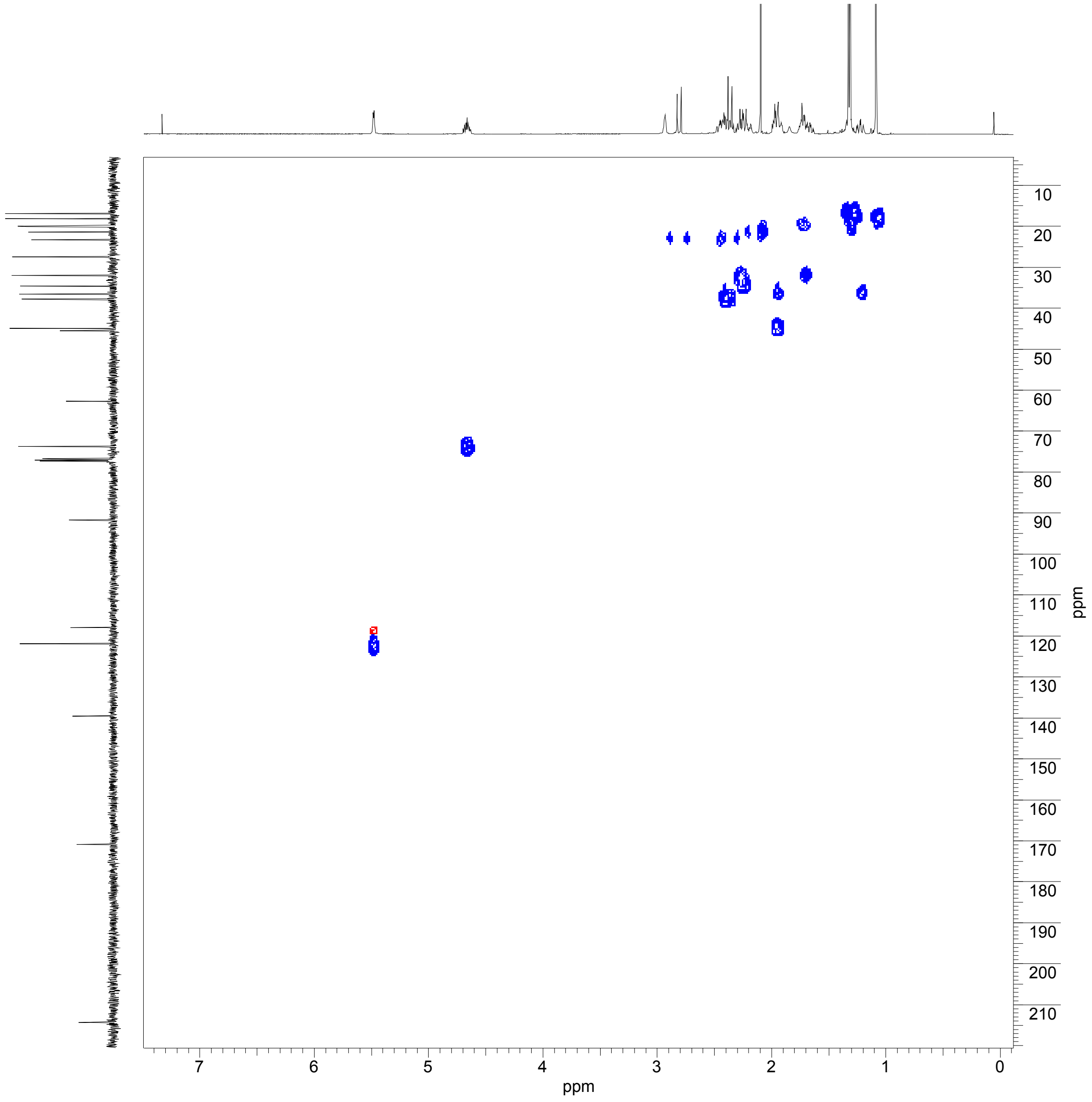

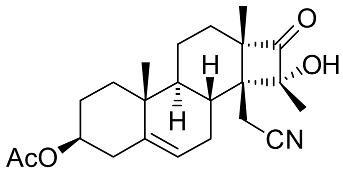

2

HMBC ($CDCl_3$)

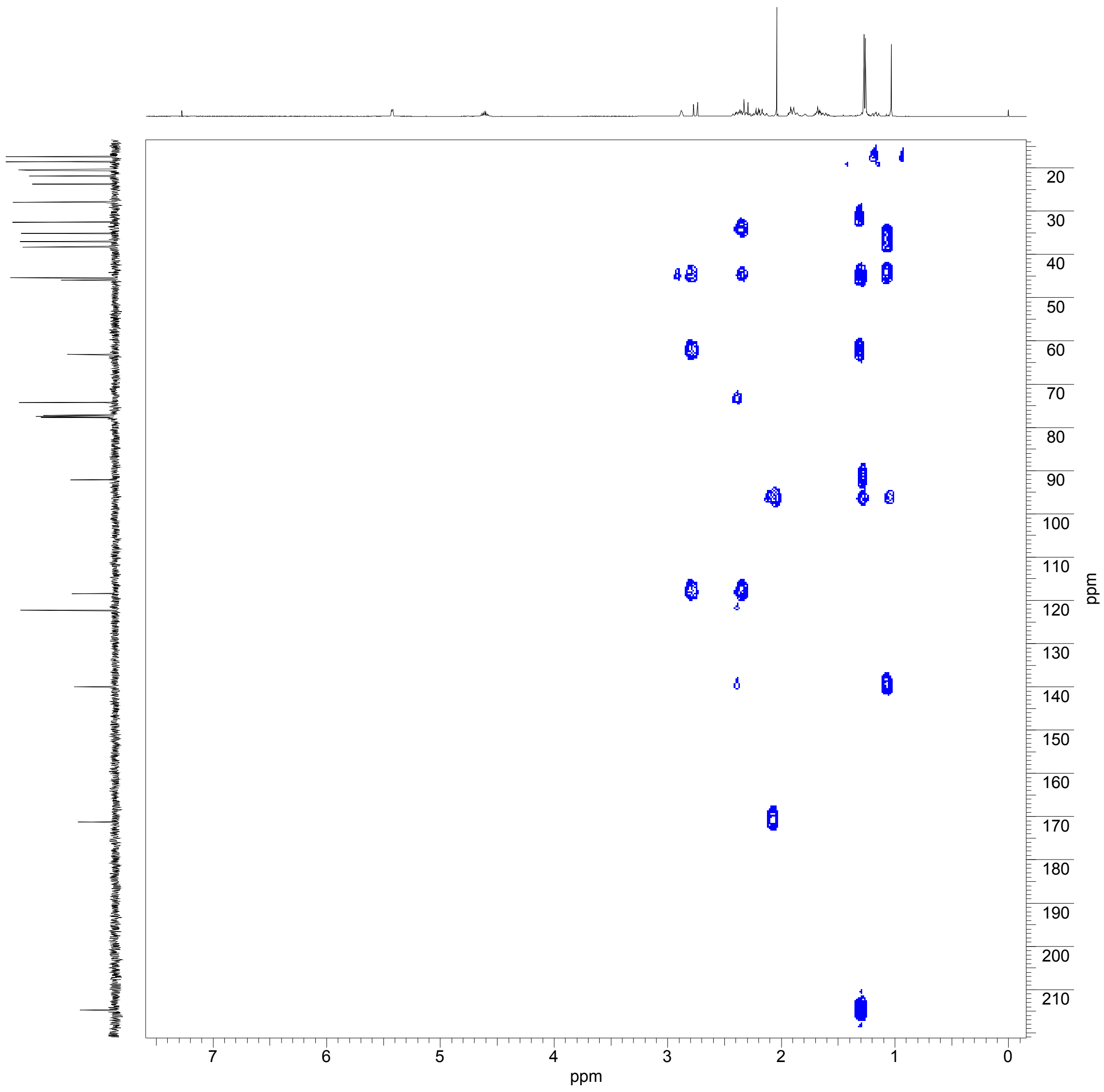

20
30
40
50
60
70
80
90
100
110
120
130
140
150
160
170
180
190
200
210
ppm
7
6
5
4
3
2
1
0
ppm

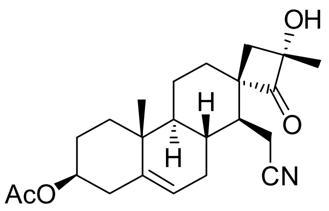

3

^{1}H NMR (500 MHz, $CDCl_3$)

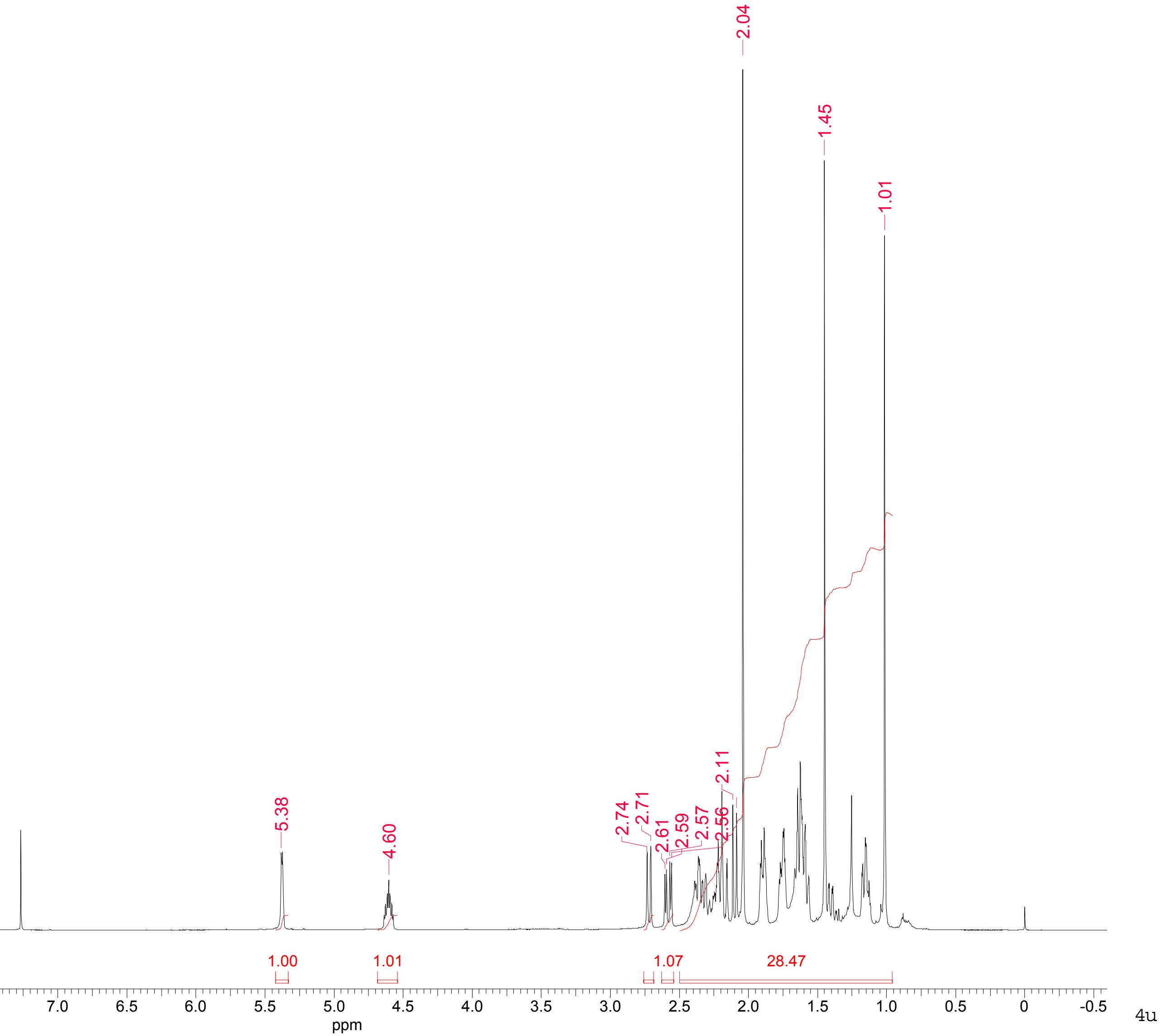

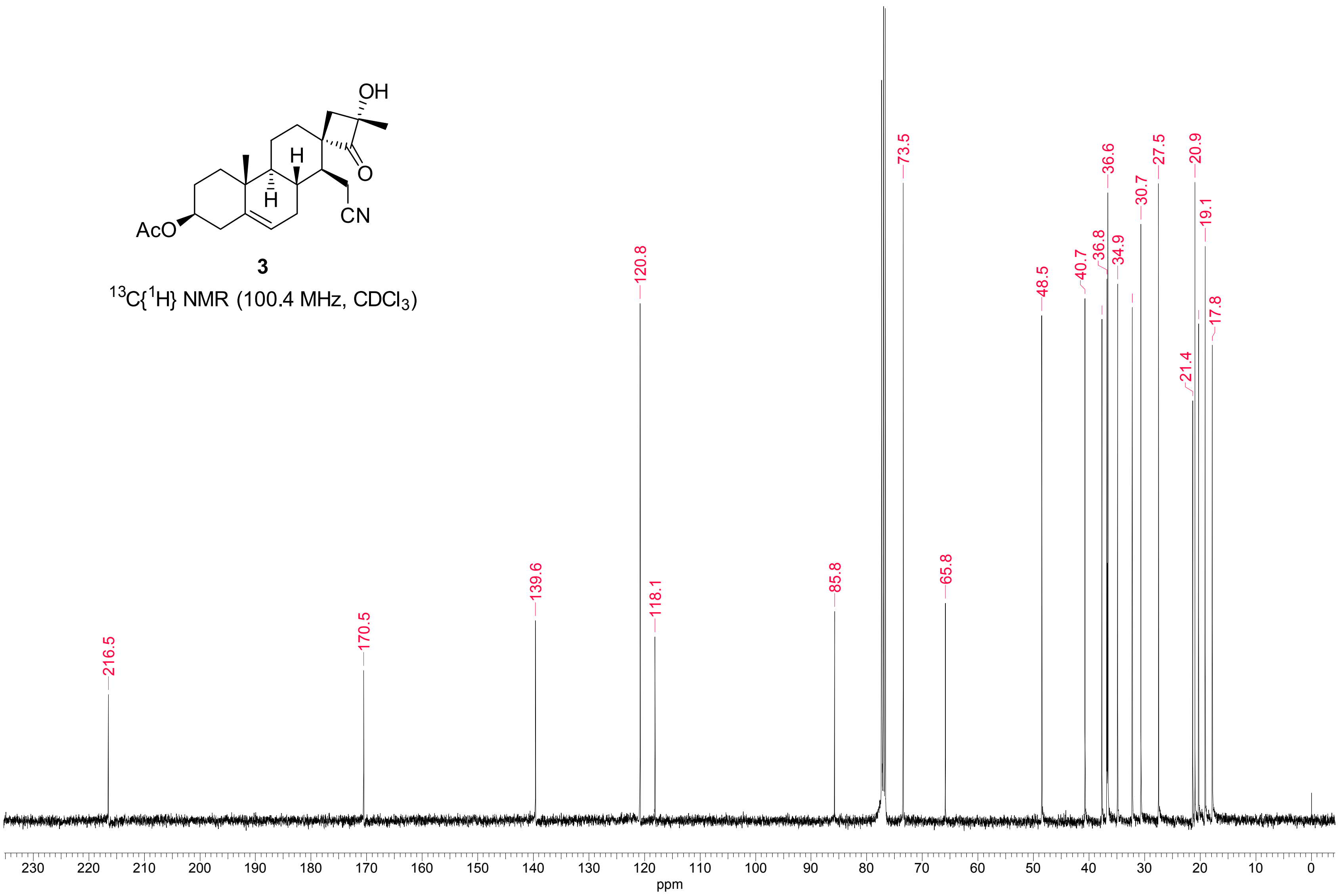

3

$^{13}C\{^{1}H\}$ NMR (100.4 MHz, $CDCl_3$)

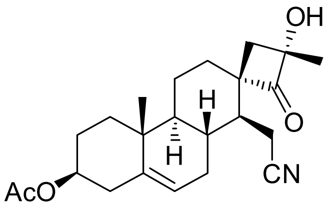

3

DEPT90 (100.4 MHz, $CDCl_3$)

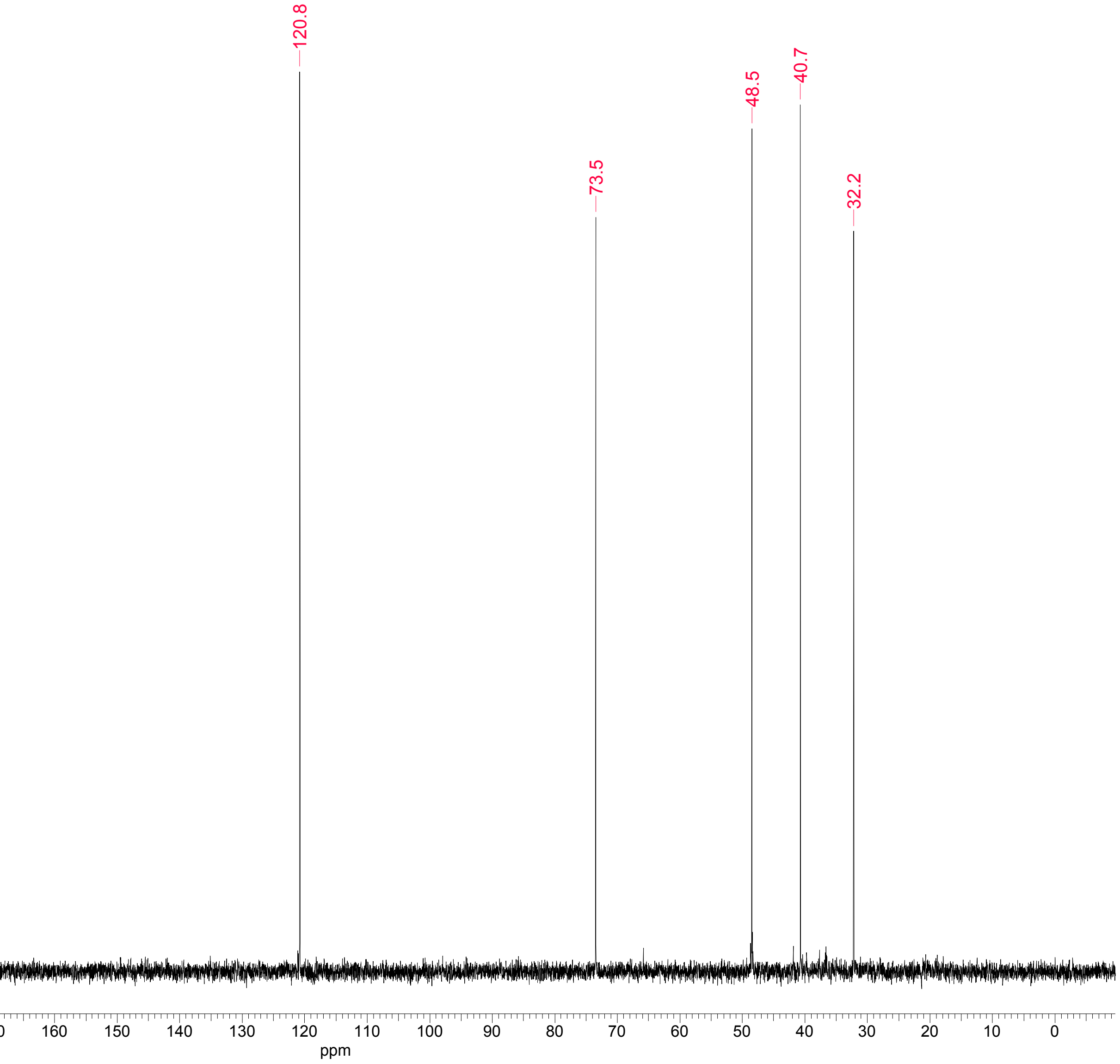

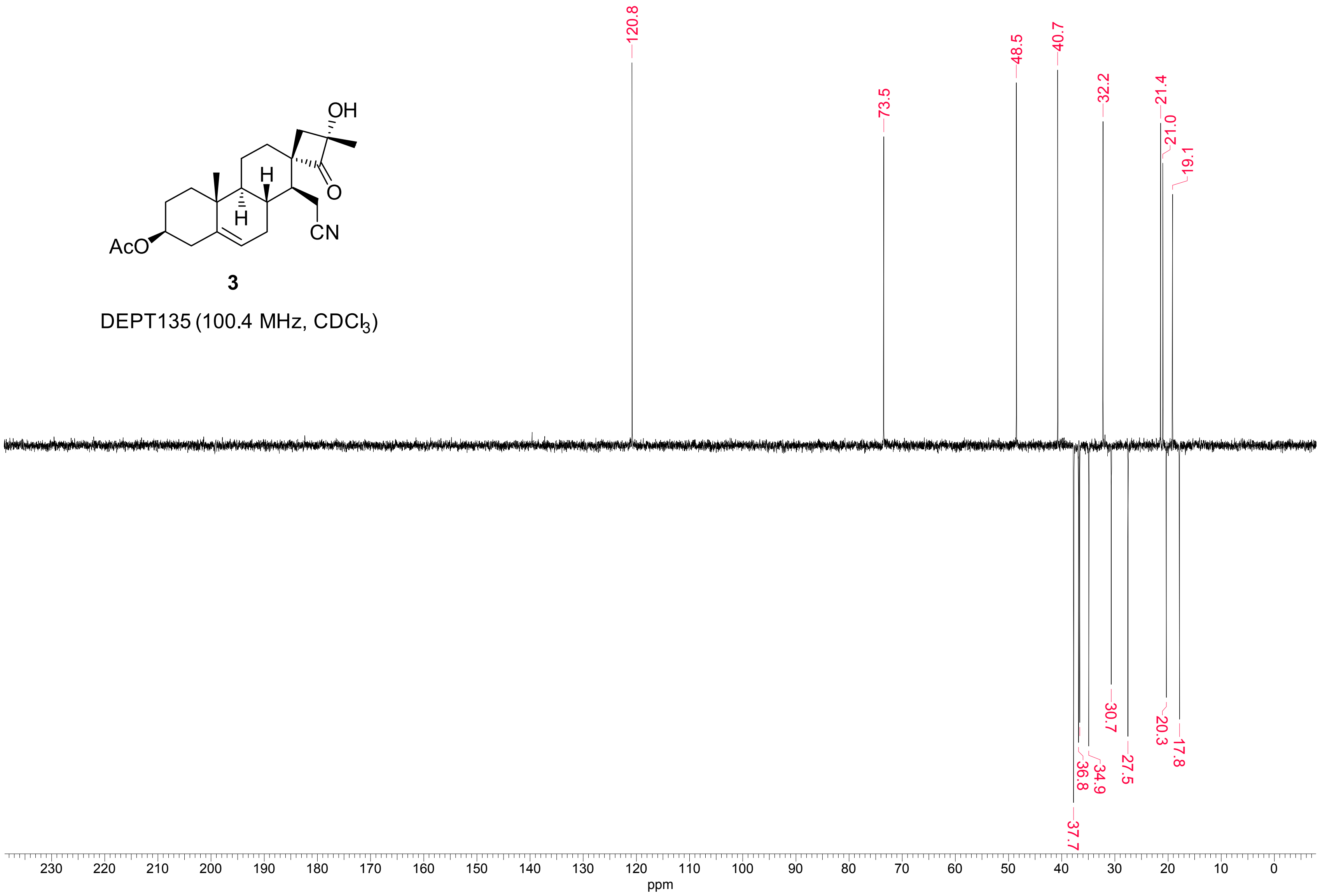

3

DEPT135 (100.4 MHz, $CDCl_3$)

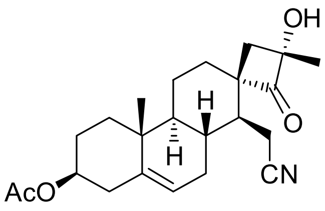

3

COSY (500 MHz, $CDCl_3$)

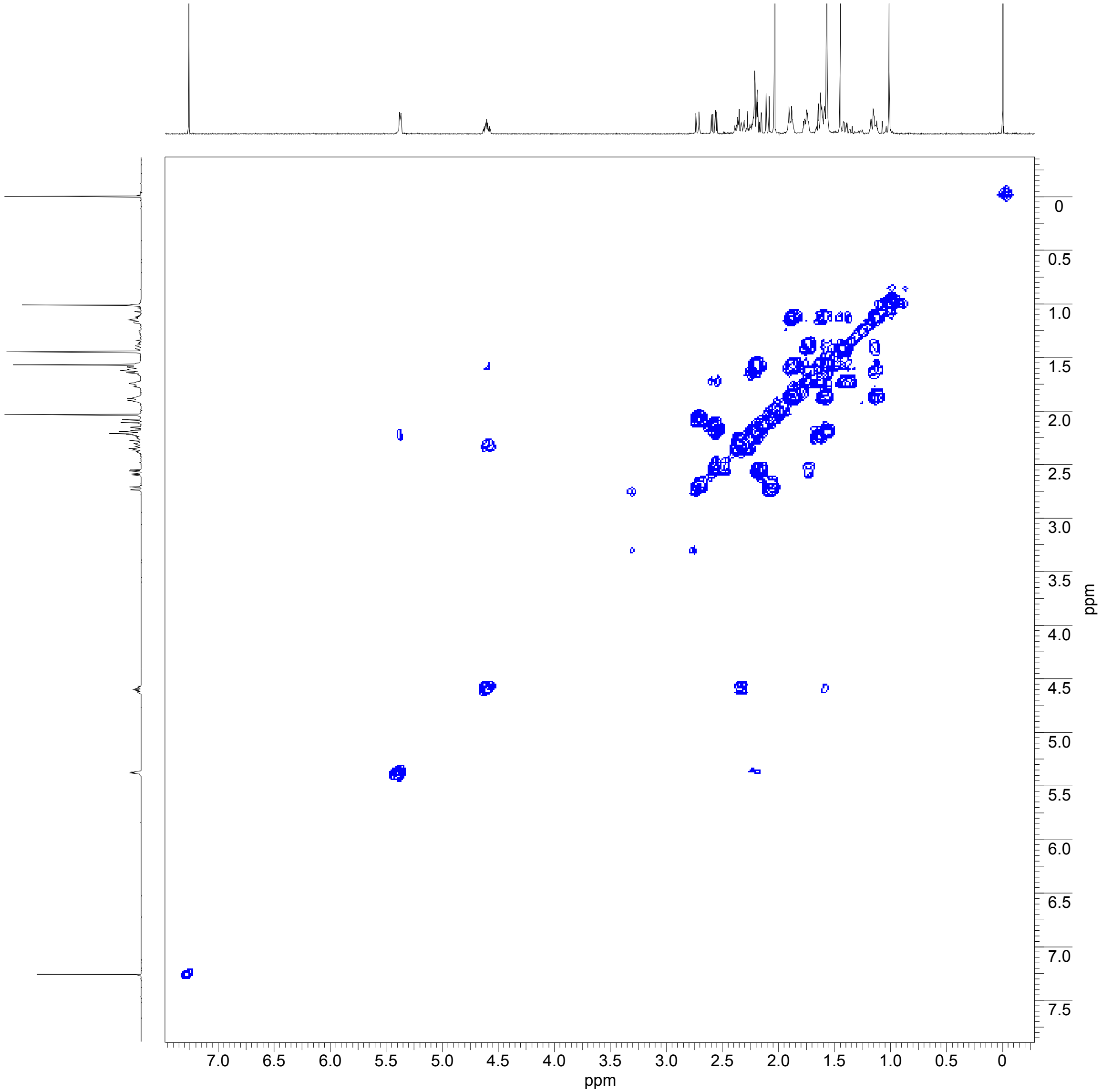

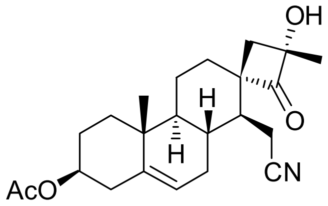

3

NOESY (500 MHz, $CDCl_3$)

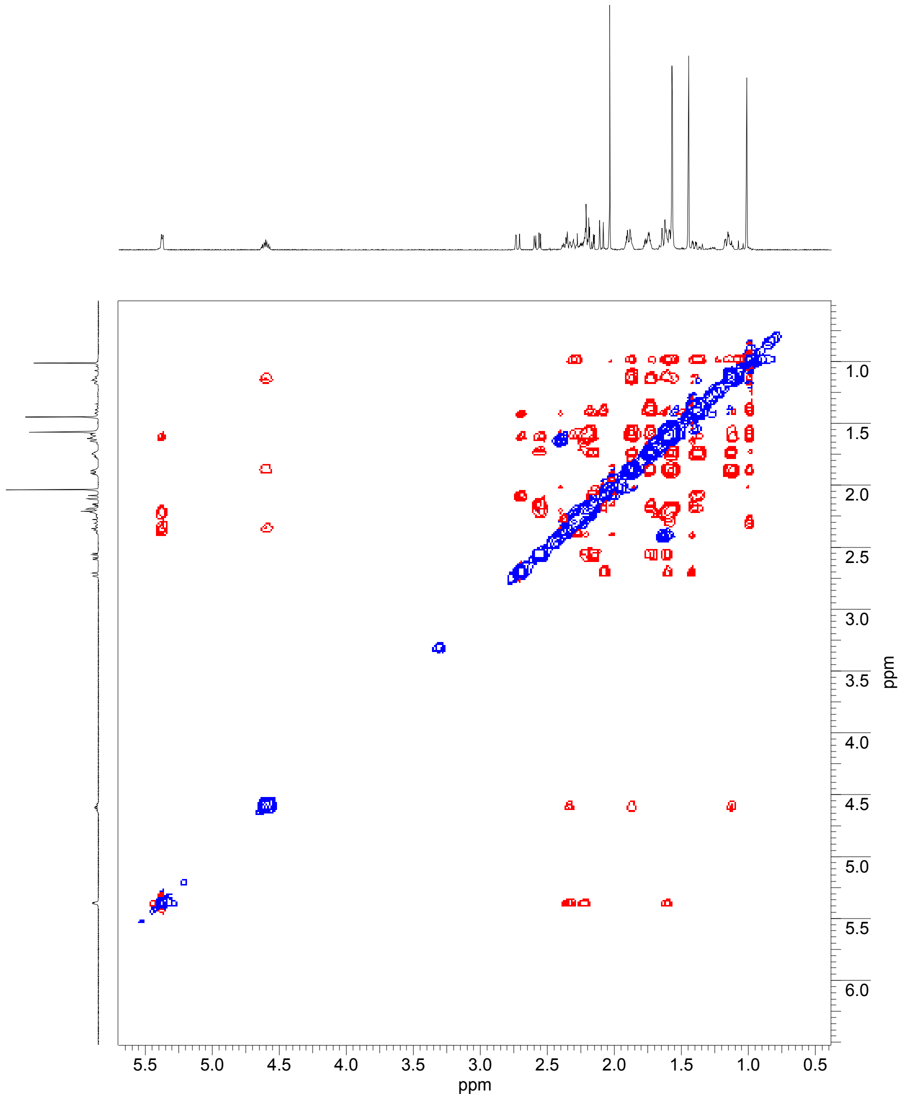

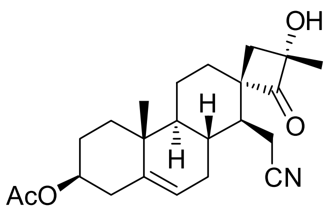

OH
H
O
H
CN
AcO

3

HSQC ($CDCl_3$)

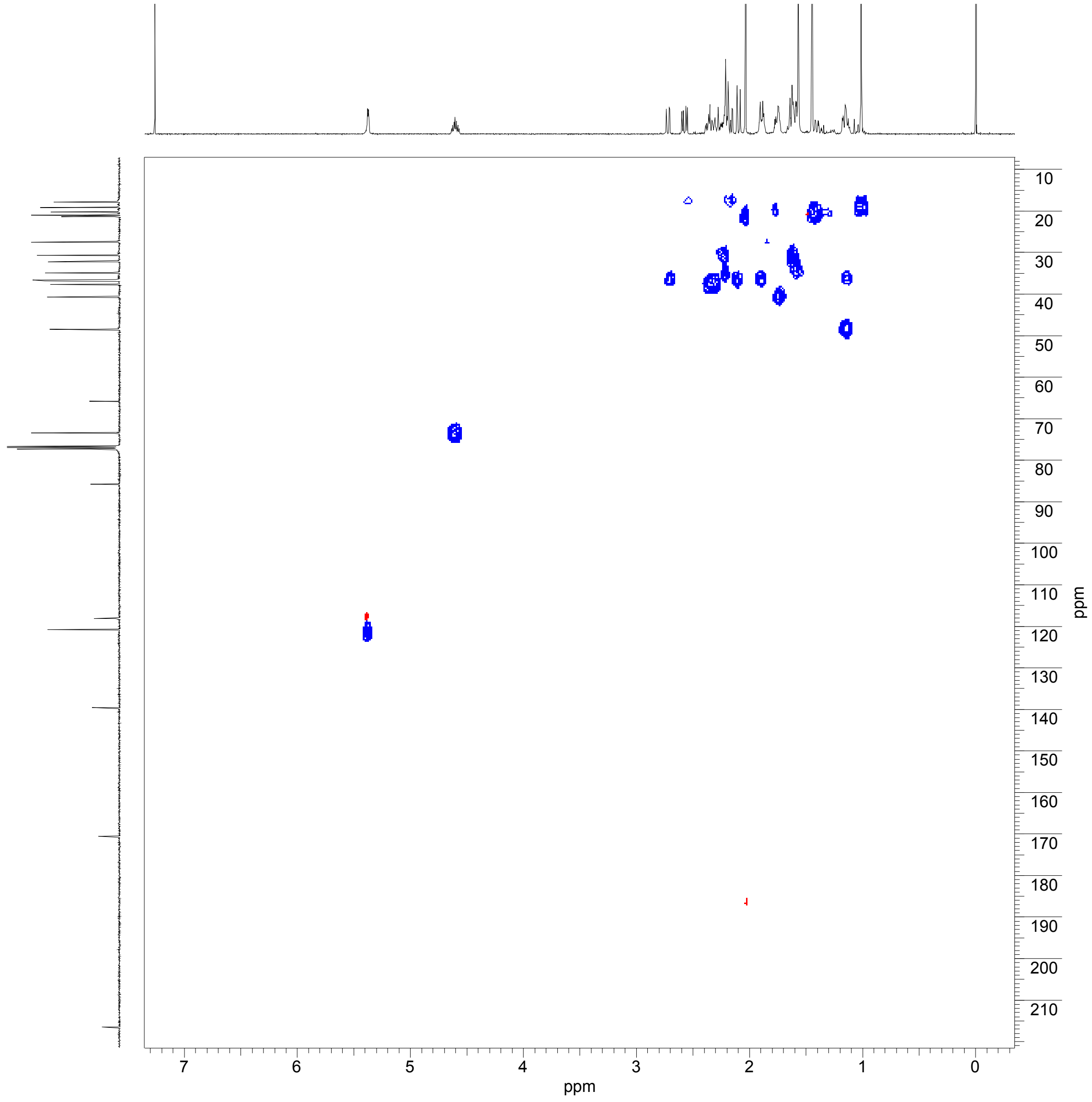

7
6
5
4
3
2
1
0
ppm
10
20
30
40
50
60
70
80
90
100
110
120
130
140
150
160
170
180
190
200
210
ppm

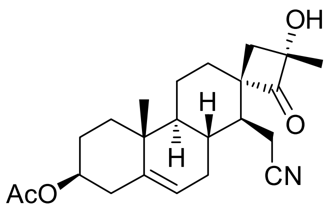

3

HMBC ($CDCl_3$)

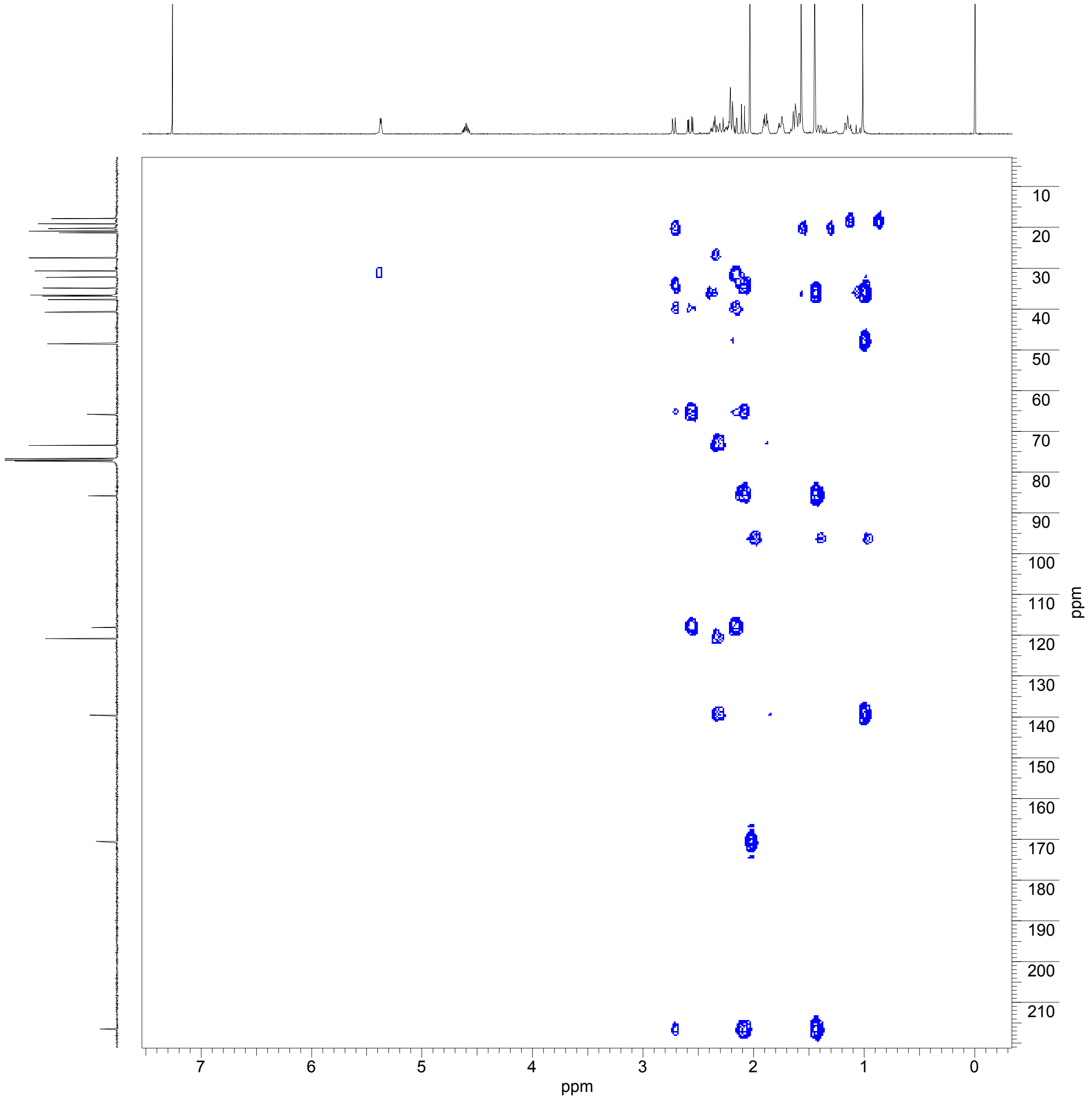

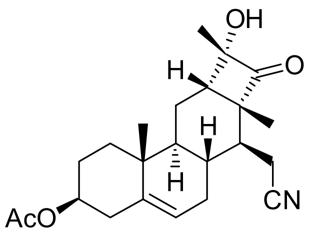

4

^{1}H NMR (500 MHz, $CDCl_3$)

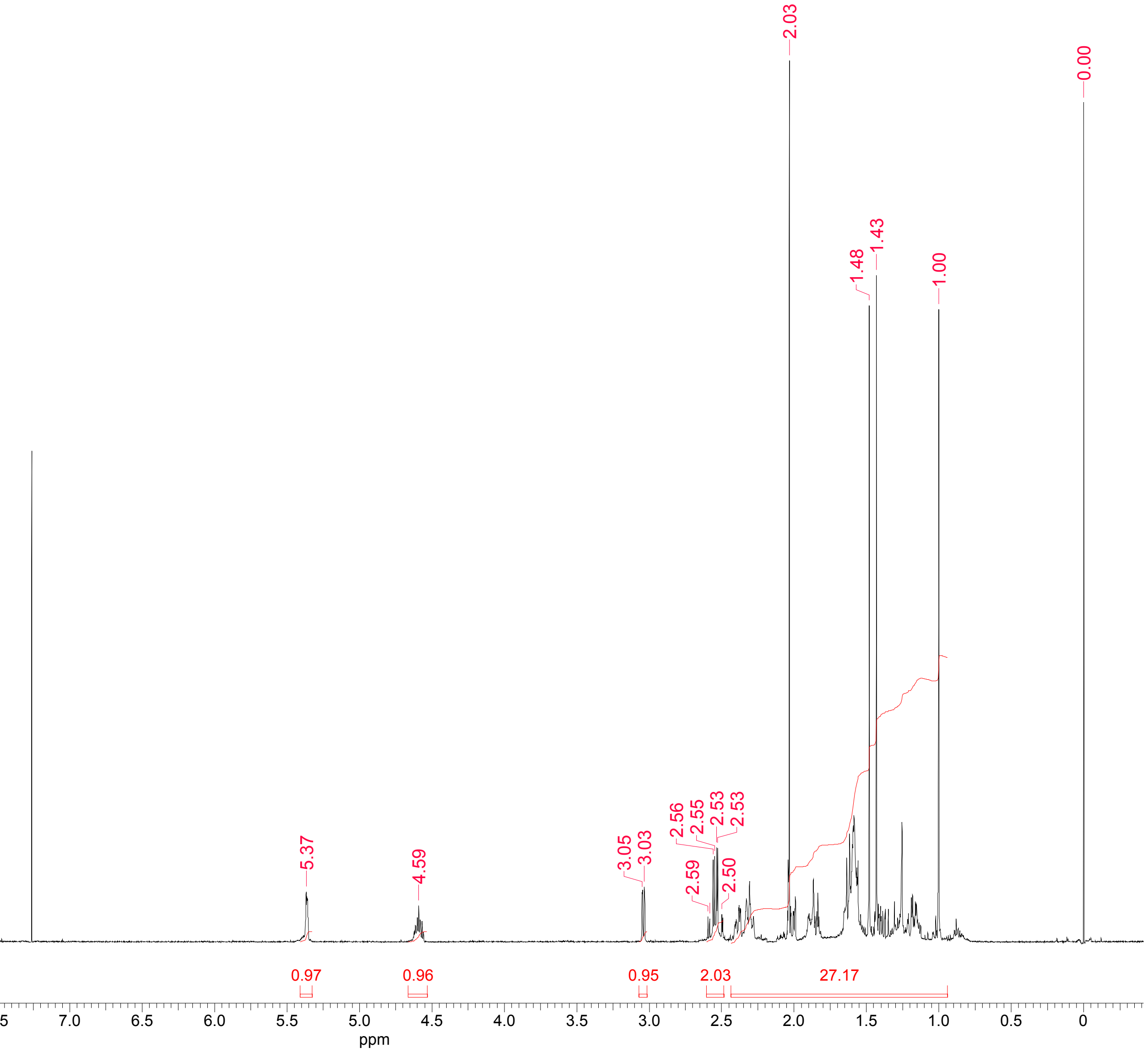

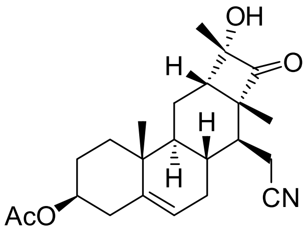

4

$^{13}C\{^{1}H\}$ NMR (125.7 MHz, $CDCl_3$)

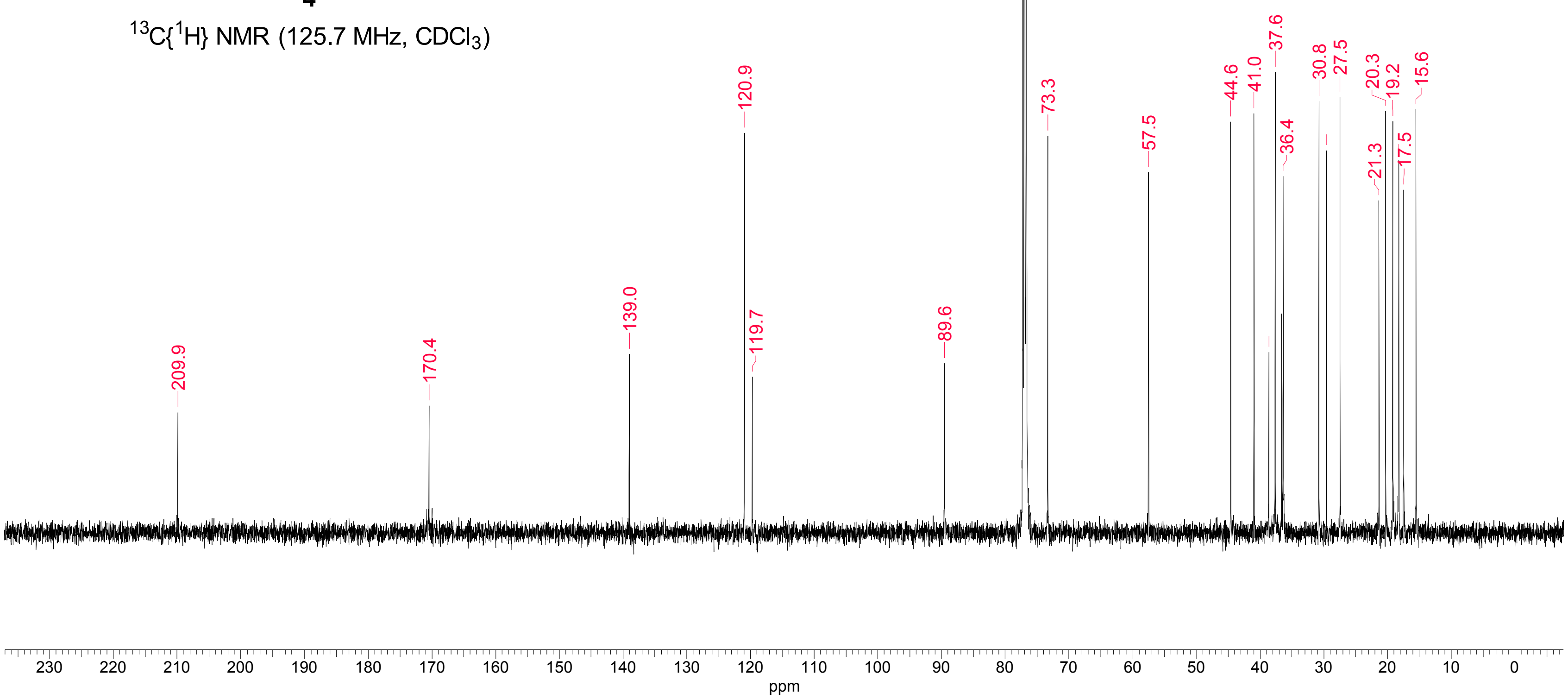

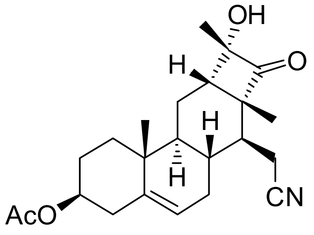

4

DEPT90 (125.7 MHz, $CDCl_3$)

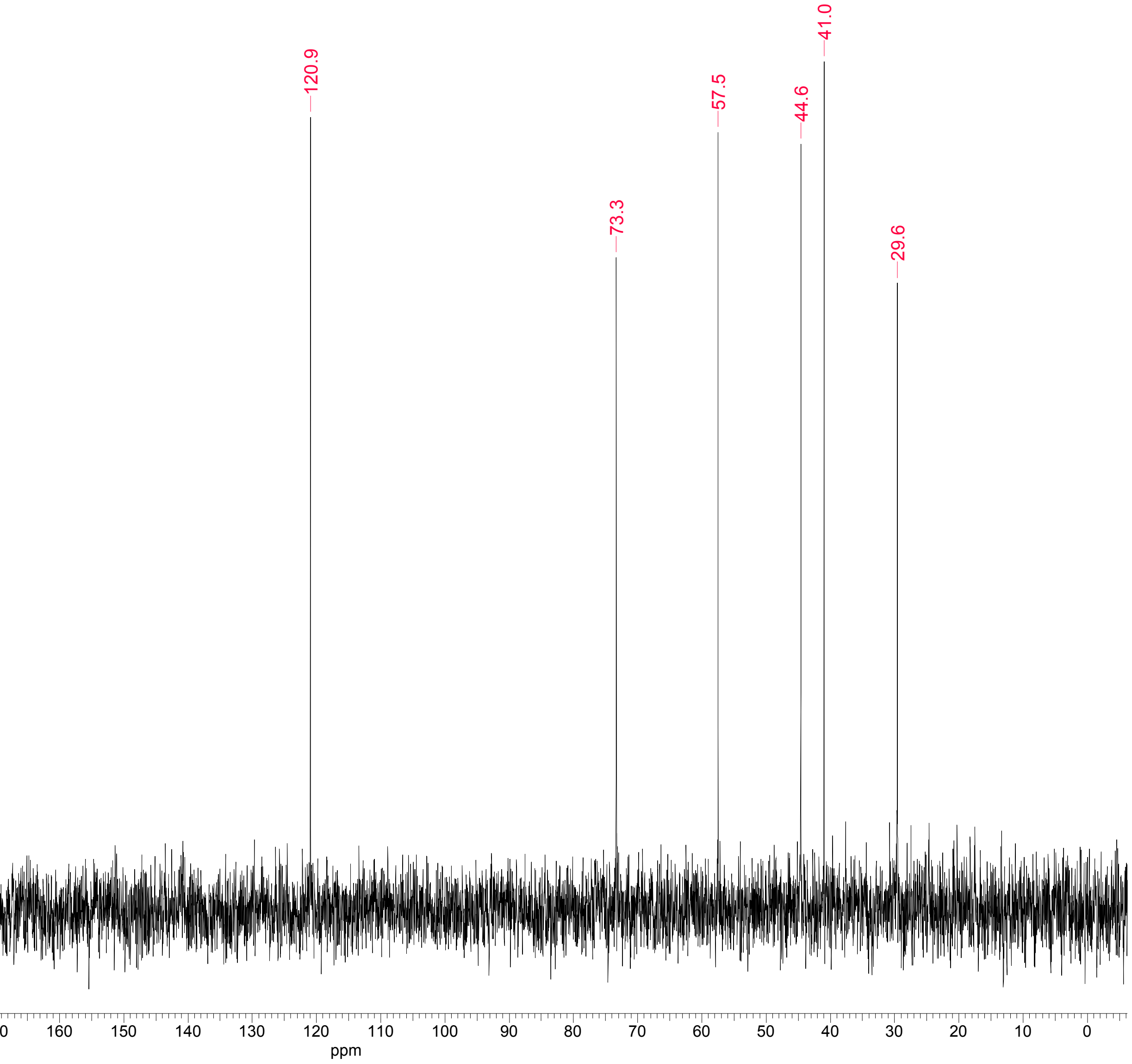

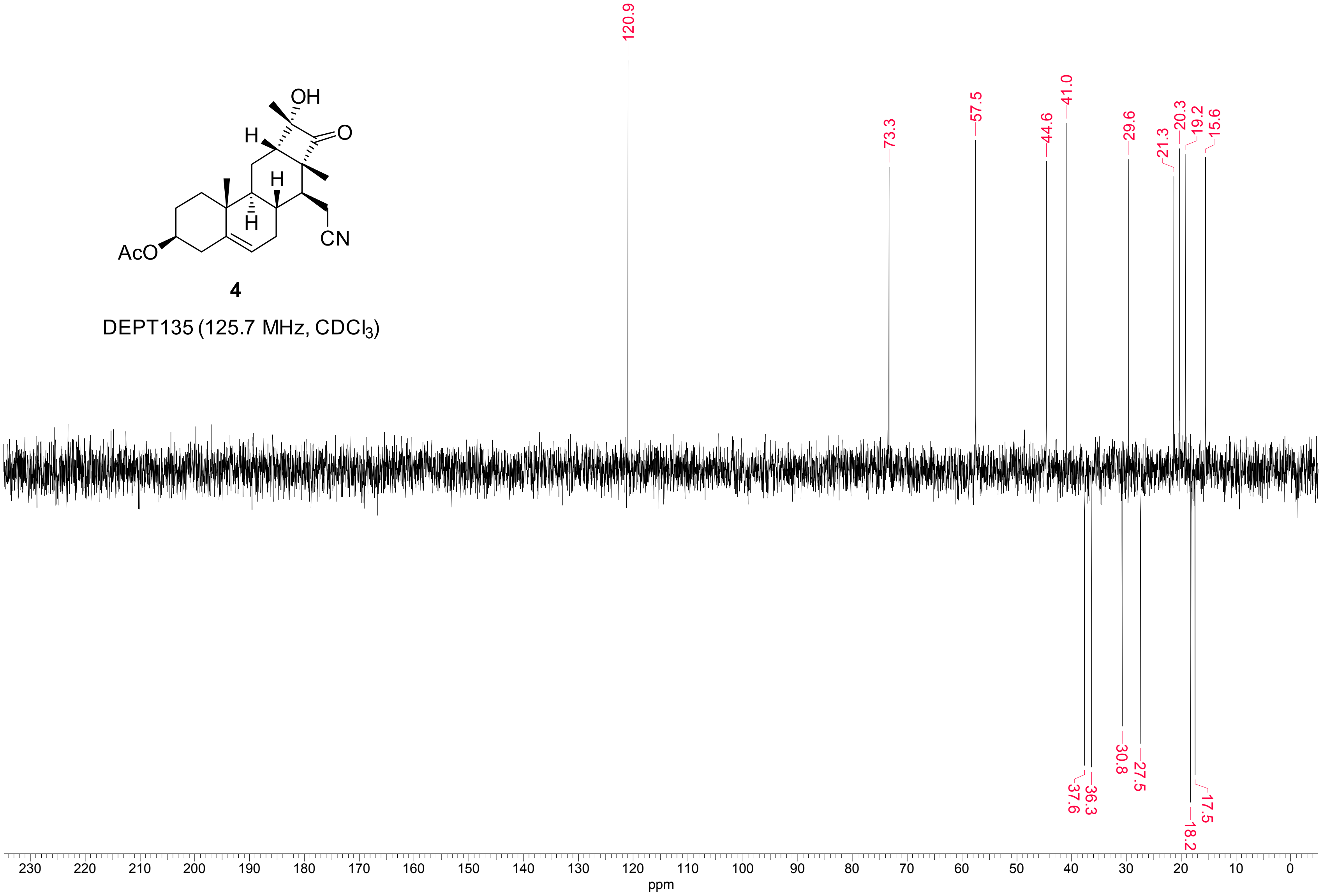
OH
O
H
H
H
AcO
CN
4
DEPT135 (125.7 MHz, CDCl3)
120.9
73.3
57.5
44.6
41.0
29.6
21.3
20.3
19.2
15.6
37.6
36.3
30.8
27.5
18.2
17.5
230 220 210 200 190 180 170 160 150 140 130 120 110 100 90 80 70 60 50 40 30 20 10 0
ppm

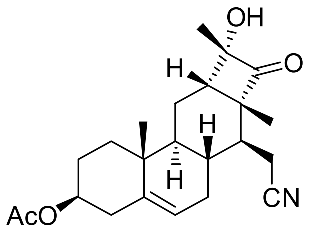

4

COSY (500 MHz, $CDCl_3$)

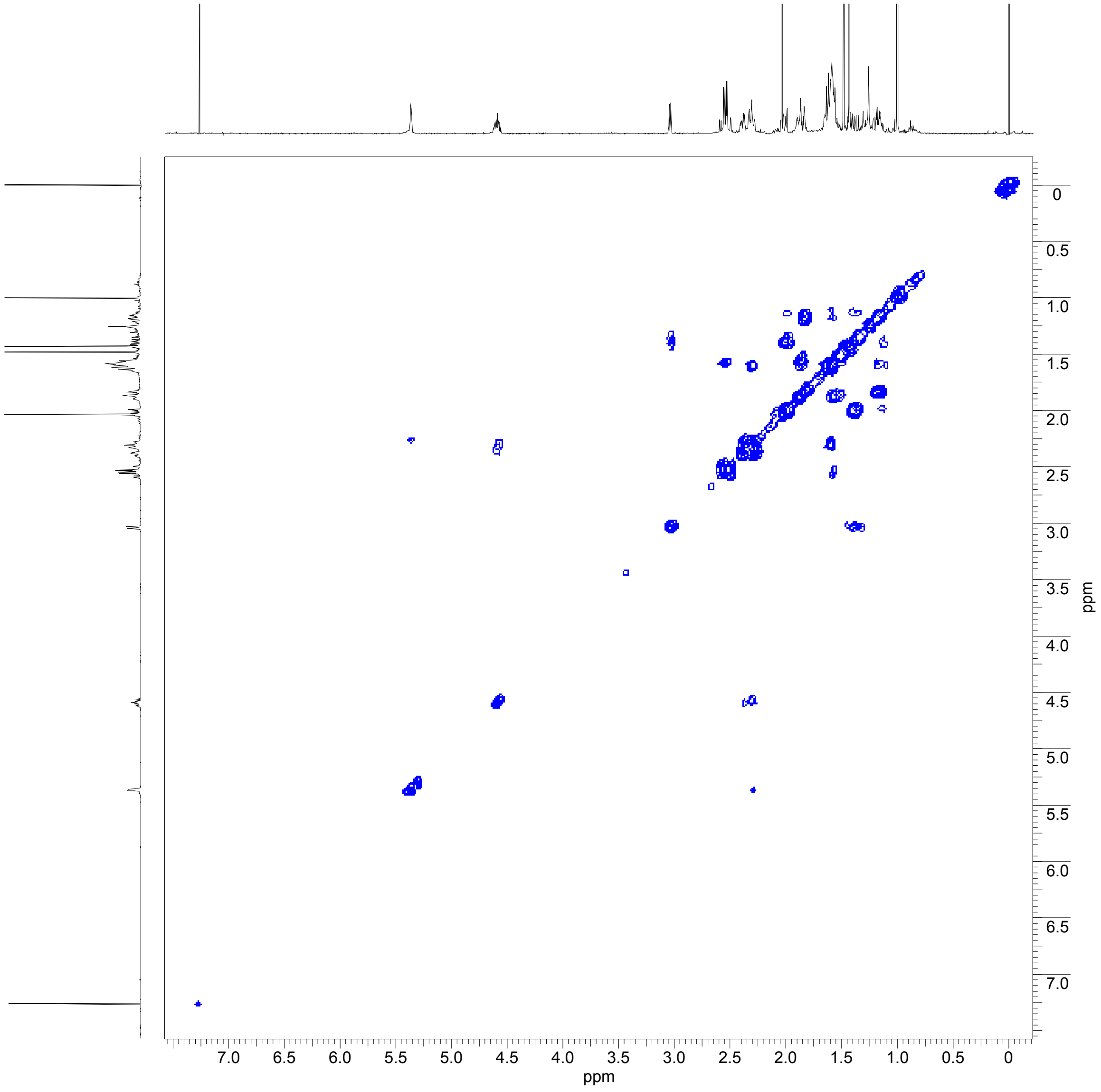

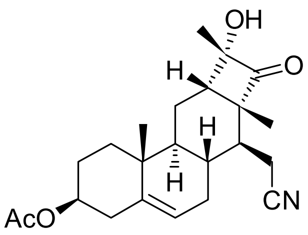

4

NOESY (500 MHz, $CDCl_3$)

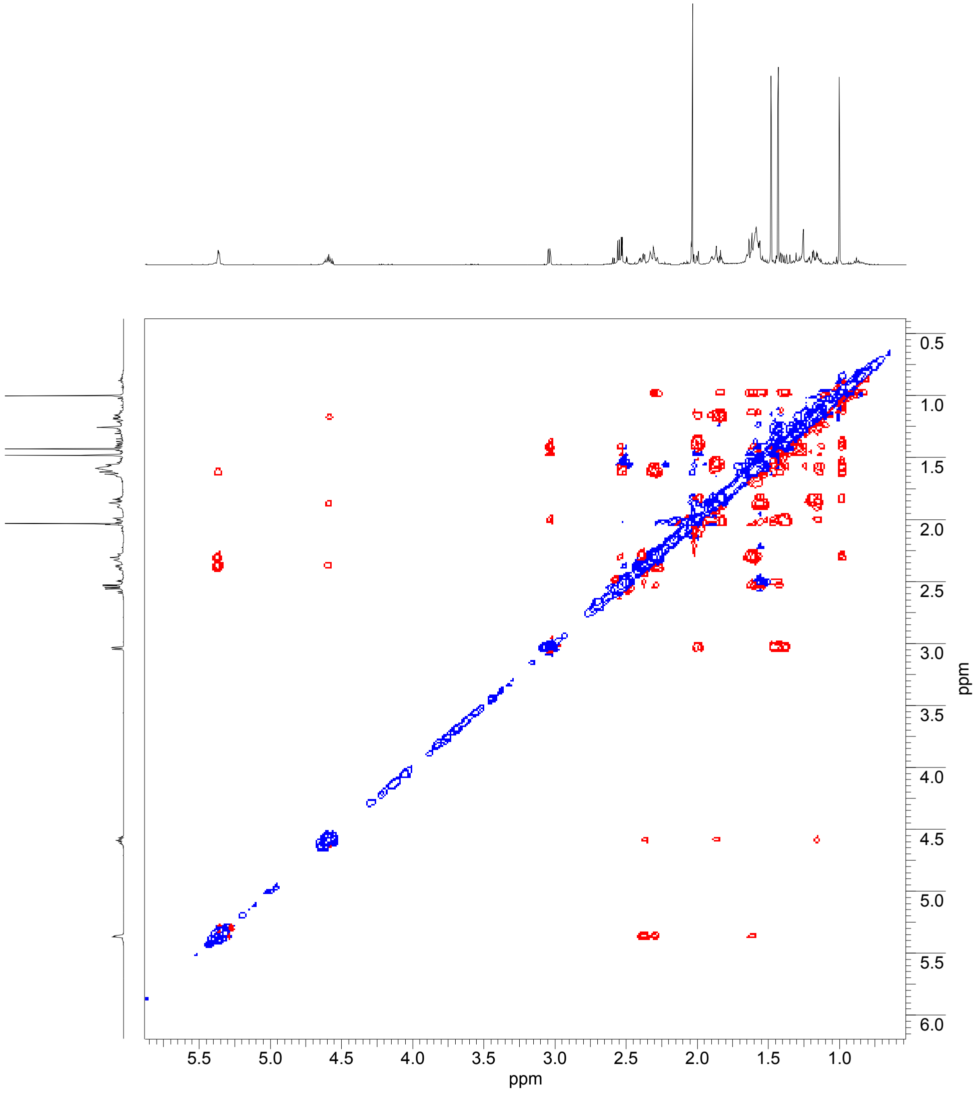

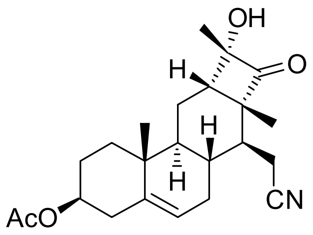
OH
H
O
H
H
AcO
CN

4

HSQC ($CDCl_3$)

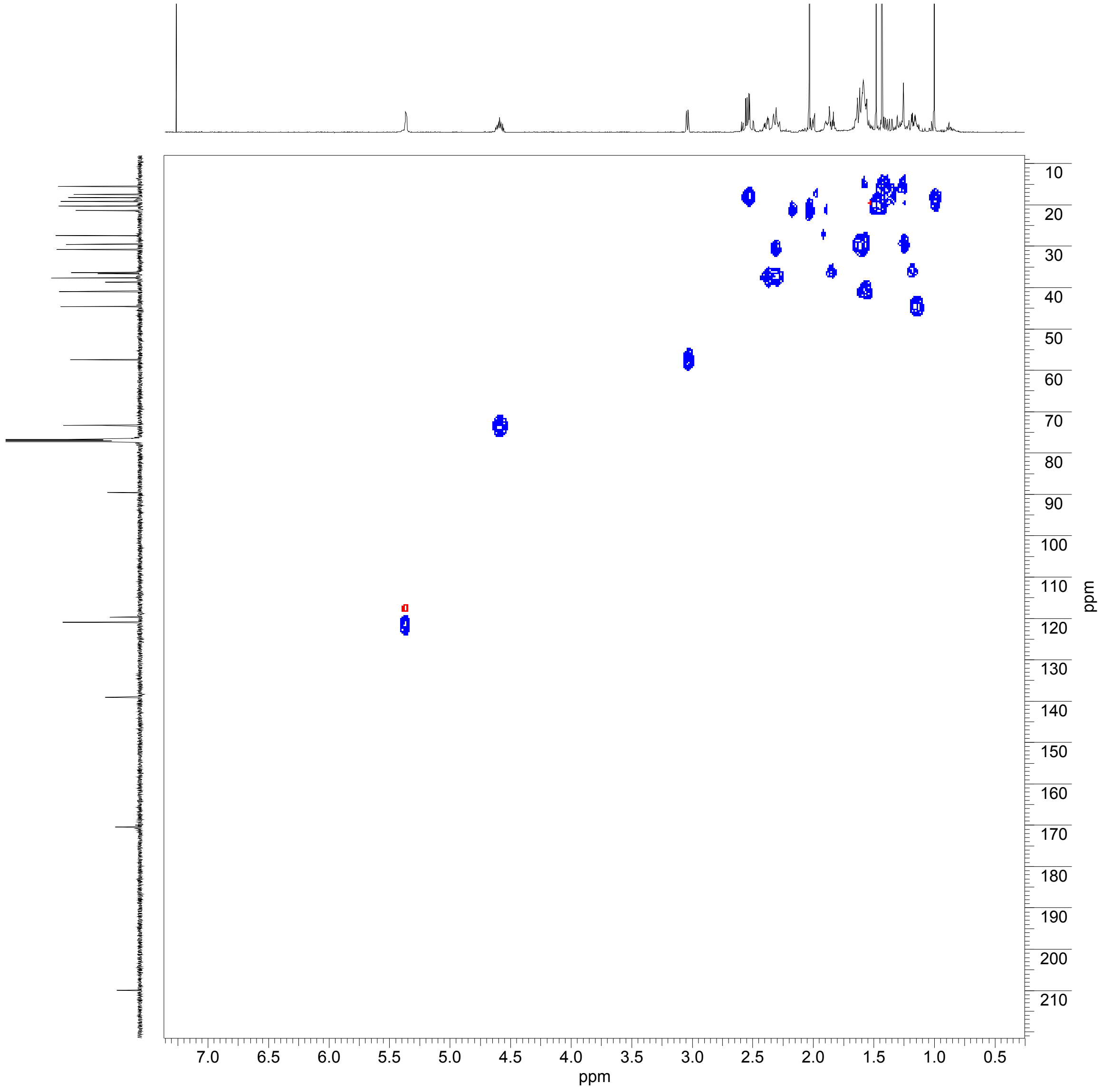
7.0 6.5 6.0 5.5 5.0 4.5 4.0 3.5 3.0 2.5 2.0 1.5 1.0 0.5
ppm
10 20 30 40 50 60 70 80 90 100 110 120 130 140 150 160 170 180 190 200 210
ppm

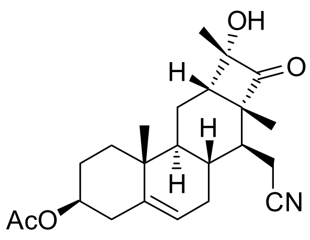

4

HMBC ($CDCl_3$)

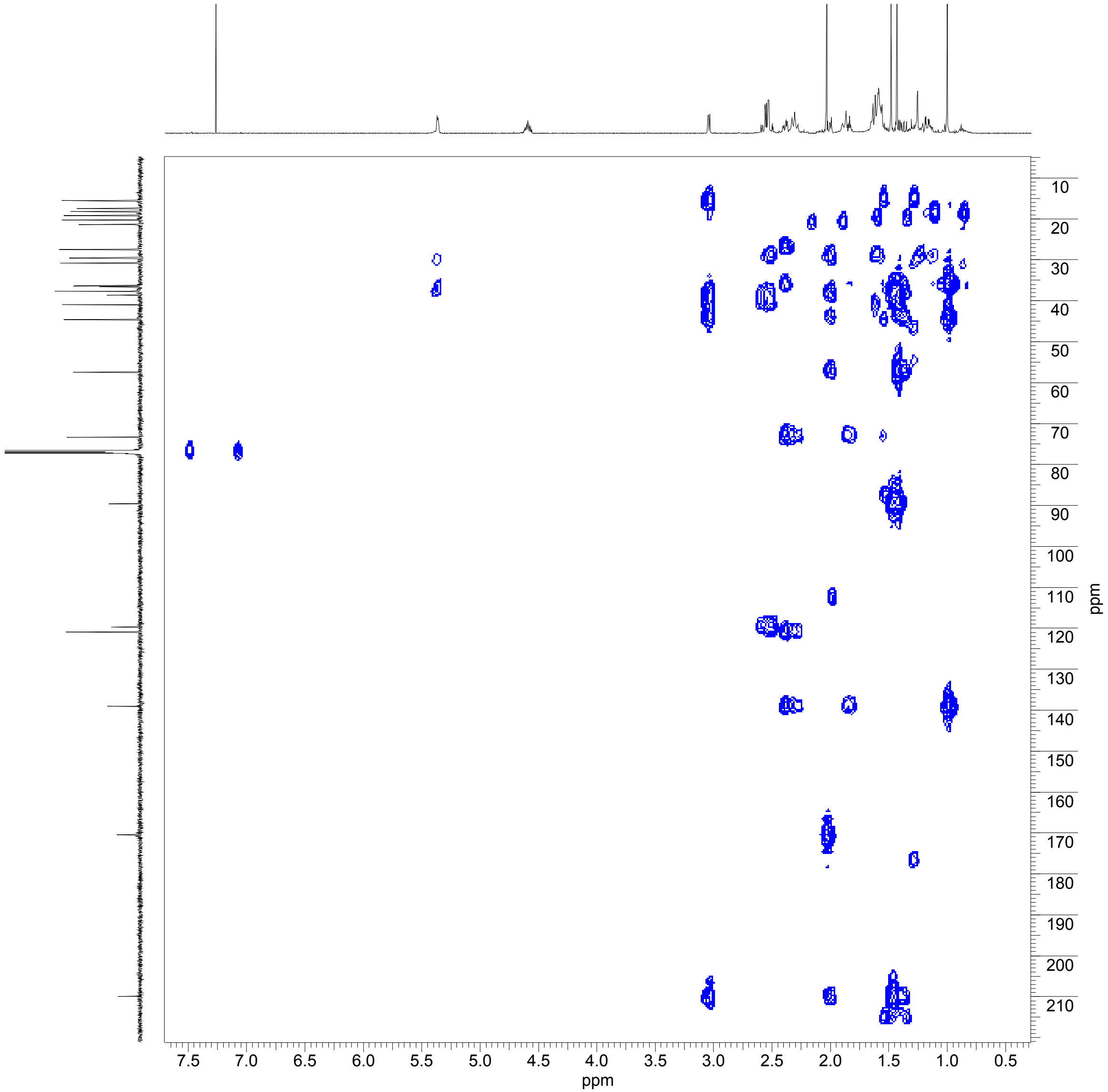

Supplement: Supplementary file 1 — jo2c01855_si_001.pdf [file jo2c01855_si_001.pdf]
